# An enhanced chemopreventive effect of methyl donor S-adenosylmethionine in combination with 25-hydroxyvitamin D in blocking mammary tumor growth and metastasis

**DOI:** 10.1038/s41413-020-0103-6

**Published:** 2020-07-22

**Authors:** Niaz Mahmood, Ani Arakelian, William J. Muller, Moshe Szyf, Shafaat A. Rabbani

**Affiliations:** 1 Department of Medicine, McGill University Health Centre, Montréal, QC H4A3J1 Canada; 2 Department of Biochemistry, McGill University, Montréal, QC H3A 1A3 Canada; 3 Department of Pharmacology and Therapeutics, McGill University, Montréal, QC H3G 1Y6 Canada

**Keywords:** Cancer, Cancer

## Abstract

Therapeutic targeting of metastatic breast cancer still remains a challenge as the tumor cells are highly heterogenous and exploit multiple pathways for their growth and metastatic spread that cannot always be targeted by a single-agent monotherapy regimen. Therefore, a rational approach through simultaneous targeting of several pathways may provide a better anti-cancer therapeutic effect. We tested this hypothesis using a combination of two nutraceutical agents S-adenosylmethionine (SAM) and Vitamin D (Vit. D) prohormone [25-hydroxyvitamin D; ‘25(OH)D’] that are individually known to exert distinct changes in the expression of genes involved in tumor growth and metastasis. Our results show that both SAM and 25(OH)D monotherapy significantly reduced proliferation and clonogenic survival of a panel of breast cancer cell lines in vitro and inhibited tumor growth, lung metastasis, and breast tumor cell colonization to the skeleton in vivo. However, these effects were significantly more pronounced in the combination setting. RNA-Sequencing revealed that the transcriptomic footprint on key cancer-related signaling pathways is broader in the combination setting than any of the monotherapies. Furthermore, comparison of the differentially expressed genes from our transcriptome analyses with publicly available cancer-related dataset demonstrated that the combination treatment upregulates genes from immune-related pathways that are otherwise downregulated in bone metastasis in vivo. Since SAM and Vit. D are both approved nutraceuticals with known safety profiles, this combination treatment may serve as a novel strategy to reduce breast cancer-associated morbidity and mortality.

## Introduction

Breast cancer is one of the most prevalent malignancies in women worldwide.^1^ Despite the recent advances in the development of anti-cancer therapeutic agents, the overall survival rate for patients with metastatic breast cancer remains poor, which highlights the need for more innovative and rational therapeutic strategies.^2^ Among the various nutraceutical agents tested for treatment of breast cancer, Vitamin D (Vit. D) showed significant promise as it decreases cell proliferation, angiogenesis, promotes cellular differentiation and apoptosis, and stimulates immune response.^3^ However, the clinical and epidemiological evidence of the anticancer effects of Vit. D remains inconclusive.^4,5^ The SUNSHINE clinical trial done on previously untreated advanced/metastatic colorectal cancer patients found that the group receiving a high dose of Vit. D, along with the standard of care chemotherapy, showed a significantly higher progression-free survival rate in comparison to the group receiving low dose Vit D. in combination with chemotherapy.^6^ The results from the recently concluded VITAL (VITamin D and OmegA-3 TriaL) study showed no statistical correlation between Vit. D supplementation and reduced incidence of cancer compared with the placebo group over a median follow-up period of 5.3 years.^7^ However, further analysis of the participants from the VITAL study who were taking Vit. D supplements for at least 2 years demonstrated a 25% reduction in cancer-related mortality. Taken together, these observations indicate the potential benefits of Vit. D supplementation alone and in combination with other well-characterized anti-cancer therapeutic agents.

Our recent studies have demonstrated that the universal methyl group donor S-adenosylmethionine (SAM) shows an antiproliferative and antimetastatic effect in the well-characterized MDA-MB-231 xenograft model of breast cancer.^8^ SAM also reduces angiogenesis and promotes apoptosis of cancer cells.^9,10^ Epigenome-wide studies in different malignancies have revealed that SAM treatment leads to hypermethylation-mediated inactivation of several key growth factors and prometastatic genes.^11,12^

Since cancer growth and metastasis requires the activity of multiple pathways, we reasoned that effective anticancer treatment strategies need to focus on coordinate targeting of several pathways. We, therefore, tested whether the combined administration of two different nutraceutical agents SAM and Vit. D, which act on different pathways critical for cancer growth and metastasis, would exhibit an enhanced anticancer and antimetastatic effect over the monotherapy with either compound on its own.

Although various types of Vit. D metabolites have shown anticancer properties, the prohormone 25(OH)D has better circulating half-life (*t*_1/2_ = 3 weeks versus 4–6 h) and lesser tendency to induce hypercalcemia than the active 1,25(OH)_2_D form.^13^ Furthermore, a recent meta-analysis demonstrated an inverse relationship between the serum levels of 25(OH)D and mortality of breast cancer patients.^14^ It has been shown that 25(OH)D can be converted to the active 1,25(OH)_2_D form locally in normal and cancerous mammary tissues by 1α-hydroxylase (CYP27B1) enzyme.^15^ Therefore, in the present study, the combined anti-cancer therapeutic potential of SAM and 25(OH)D was assessed in vitro using a panel of breast cancer cell lines. For in vivo studies, we used the well-established transgenic MMTV-PyMT (mouse mammary tumor virus promoter-driven polyoma middle T oncoprotein) mouse model of breast cancer to monitor mammary tumor emergence, growth, and lung metastasis and a syngeneic model using PyMT-R221A cells to evaluate the effect on skeletal colonization by breast tumor cells.^16^ Our results show that combination treatment significantly delays mammary tumor emergence, reduces tumor volume, and metastasis in comparison with either monotherapy without showing any adverse effects.

## Results

### Combination of SAM and 25(OH)D suppresses cell proliferation and clonogenic survival potential in vitro

**Fig. 1:**
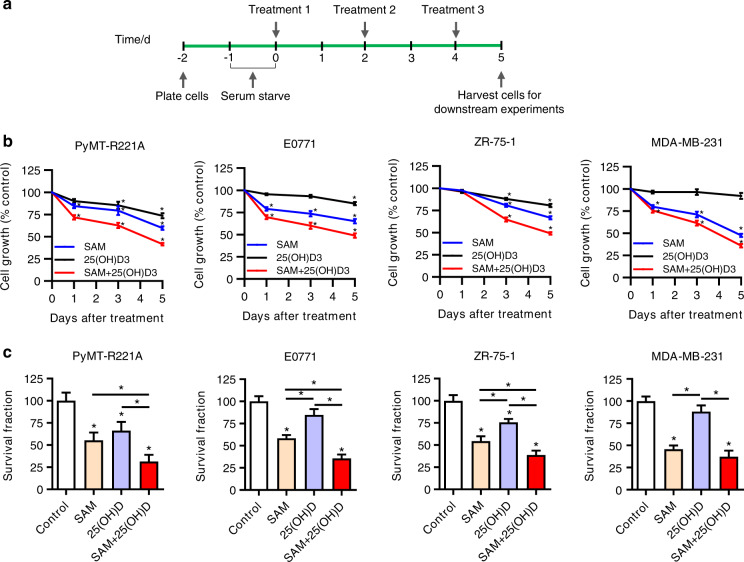
Effect of SAM, 25(OH)D, and their combination in vitro. **a** Schematic of the in vitro treatment protocol. **b** Human (ZR-75-1, MDA-MB-231) and murine (PyMT-R221A, E0771) breast cancer cells were treated with vehicle alone control, SAM (200 µmol·L^−1^) or 25(OH)D (100 nmol·L^−1^) alone and SAM + 25(OH)D every other day; and cell number was determined using Coulter counter on days 1,3, and 5 post-treatment. Results are shown as the mean ± SEM from at least five independent experiments. Significant differences from the control groups in each cell lines were determined using ANOVA followed by *post hoc* Tukey’s test and are represented by asterisks. **c** Following treatment, 5 × 10^3^ cells from the control and different treatment groups were subjected to clonogenic survival assay. The culture media was refreshed every 3–4 days for a period of about 2 weeks, the cells were then stained with crystal violet, and the total number of colonies was counted under the microscope. Results are shown as the mean ± SEM of at least five independent experiments. Significant differences were determined using ANOVA followed by *post hoc* Tukey’s test and are represented by asterisks

Since cancer is a disease of uncontrolled cell proliferation and survival,^17^ a panel of human (ZR-75-1, MDA-MB-231) and murine (PyMT-R221A, E0771) breast cancer cell lines with different levels of endogenous Vit. D receptor expression (Supplementary File 1, Fig. S1) was used to examine the possible growth inhibitory effects of SAM and 25(OH)D combination in vitro. Using a treatment protocol shown in Fig. 1a, we found that single-agent treatment with SAM (200 μmol·L^−1^) resulted in a significant reduction in cell proliferation as compared with the control cells treated with vehicle alone during the same period (Fig. 1b). Treatment with 25(OH)D (100 nmol·L^−1^) monotherapy also caused significant repression in the growth properties of PyMT-R221A, E0771, and ZR-75-1 cells, results which are consistent with previous reports.^18,19^ Notably, the anticancer effects on the growth properties were more pronounced in the cells treated with the combination of SAM and 25(OH)D (Fig. 1b). Next, we calculated the coefficient of drug interaction (CDI) to characterize whether the nature of the interaction between the SAM and 25(OH)D are synergistic, additive, or antagonistic using the following equation: CDI = AB/(A × B).^20,21^ The CDI values indicated that the combination of SAM and 25(OH)D shows an additive effect to moderately synergistic effect at the doses used in this study (Supplementary File 1, Fig. S2). We also treated normal human breast epithelial cells (HBEC) with SAM, 25(OH)D, and SAM + 25(OH)D combination and found no significant change in the viability of HBEC cells treated with different agents when compared with the vehicle-treated control cells, suggesting that the treatments are not toxic at the drug concentration used in vitro (Supplementary File 1, Fig. S3).

Next, to further test the antiproliferative effect, we examined the impact of different treatments on the colony-forming ability of these cell lines using a clonogenic survival assay. Our data showed that the SAM + 25(OH)D combination decreases the colony-forming potential of all four cells compared with the vehicle-treated controls as well as those treated with either SAM or 25(OH)D alone (Fig. 1c). Taken together, these results suggest that the combined use of SAM and 25(OH)D may serve as an effective strategy for breast cancer treatment.

### Combination of SAM and 25(OH)D delays mammary tumor development and attenuates tumor growth and lung metastasis in transgenic MMTV-PyMT mice

**Fig. 2:**
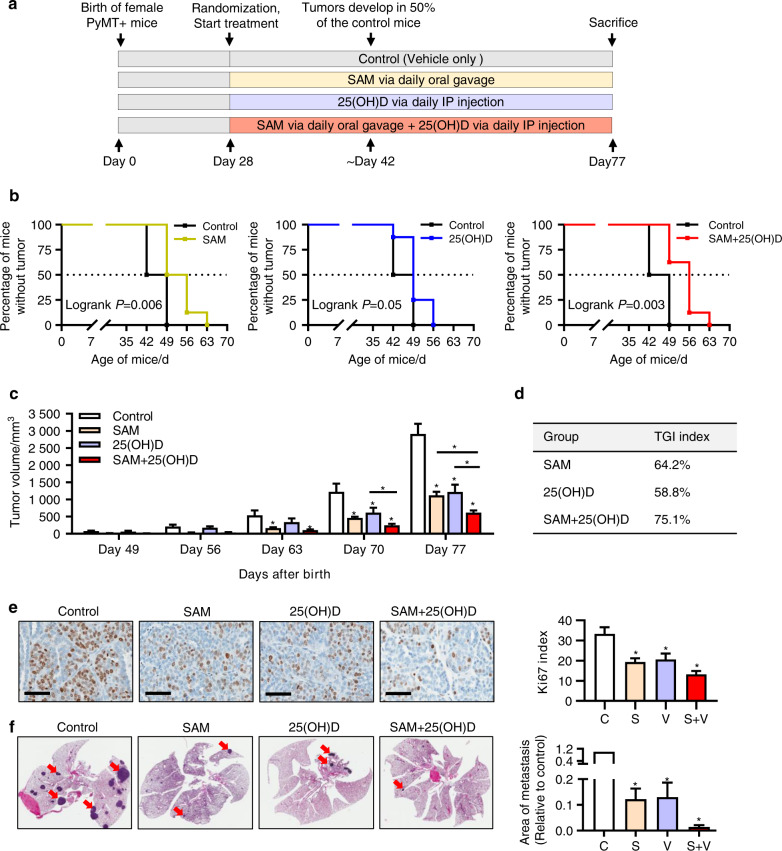
Effect of SAM, 25(OH)D, and their combination on mammary tumor emergence, growth, and lung metastasis in MMTV-PyMT transgenic female mice. **a** Schematic representation of treatment protocol for the transgenic MMTV-PyMT mice. Briefly, female MMTV-PyMT mice were treated with vehicle alone as control, SAM (160 mg·kg^−1^ per day) by daily oral gavage, 25(OH)D (40 ng·kg^−1^ per day) by daily intraperitoneal (i.p) injection, and SAM + 25(OH)D in combination from day 28 (week 4) after birth until the experimental endpoint at day 77 (week 11) when the animals were all sacrificed (*n* = 8 animals per group) and different tissues were collected for downstream experiments. **b** Kaplan–Meier curve showing the percentage of mice no tumor in control and different treatment groups, separately (*n* = 8 animals per group). **c** Tumor volumes were measured at weekly intervals using calipers and are shown as a bar graph. Results are shown as the mean ± SEM of eight animals per group. Significant differences were determined using ANOVA followed by *post hoc* Tukey’s test and are represented by asterisks. **d** Tumor growth inhibition (TGI) index at sacrifice was calculated using the formula described in ‘Materials and Methods’ and represented in a tabular format. **e** The formalin-fixed histologic sections of the mammary tumors from control and different treatment groups were probed with the antibody for ki67 proliferation marker, and representative images of the ki67 positive cells (brown color staining) is shown for different groups [scale bar size = 60 μm]. The percentage of ki67 positive cells was determined and plotted as bar graphs (*n* = 4 animals per group). **f** For the evaluation of lung metastasis, formalin-fixed histologic sections of the whole lung tissue sections from control and treated animals were stained with Haemotoxylin and Eosin (H&E) (left panel), and the relative area of metastases was quantified using the Fiji plugin (ImageJ) (right panel). The metastatic sites on the lung show darker staining patterns, as indicated by red arrowheads. Results are shown as mean ± SEM (*n* = 4 per group). The metastatic area in the control group was set to 1 for the statistical analysis for the bar graph. Here, C = control, S = SAM, V = 25(OH)D, S + V = SAM + 25(OH)D treated group

To assess whether the combination treatment has any effect on mammary tumor emergence and volume, we used the well-characterized transgenic MMTV-PyMT mice (in FVB background) that mimic the step-wise progression of breast cancer in humans.^22^ Female MMTV-PyMT mice were randomized on day 28 after birth to four different treatment groups: vehicle-treated controls, SAM (160.0 mg·kg^−1^ per day) via oral gavage, 25(OH)D (40.0 ng·kg^−1^ per day) by intraperitoneal injection, and combination of SAM and 25(OH)D at the same concentrations (Fig. 2a).

Control and experimental groups of animals were monitored for the appearance of both axillary (anterior) and inguinal (posterior) mammary tumors from day 35 after birth. We found that the vehicle-treated control mice spontaneously developed palpable mammary tumors at around 42 days of age while a substantial delay in tumor emergence was observed for all three treatment groups (Fig. 2b). The median value for tumor emergence in the control group was day 45.5 after birth, which was delayed to day 49.5 and 52.5 in the 25(OH)D (log-rank *P* = 0.05) and SAM (log-rank *P* = 0.006) monotherapy treated groups, respectively (log-rank *P* = 0.021) (Fig. 2b). A further delay in tumor appearance was observed in groups treated with SAM + 25(OH)D combination (log-rank *P* = 0.003), with a median of 56 days.

Next, the total mammary tumor volume (sum of individual axillary and inguinal tumor volumes for each of the animals as described in ‘Materials and Methods’) was measured for each animal from day 49 until sacrifice on day 77 (Fig. 2c). Out data indicated a significant reduction in primary mammary tumor volumes in all three treatment groups (Fig. 2c, d). However, these effects were more pronounced in the SAM + 25(OH)D treated animals suggesting enhanced therapeutic potential of the combination treatment compared with the single-agent monotherapies in vivo.

One of the known adversities of long-term administration of Vit. D is the possibility to develop hypercalcemia.^23^ Therefore, at the time of sacrifice, serum from all animals was collected and levels of calcium and other biochemical parameters were examined. No significant difference in serum calcium or any other biochemical parameter was seen between control and experimental animals (Supplementary File 1, Table S1), suggesting that SAM and 25(OH)D had no adverse effects in vivo at these doses. In addition, no significant difference in the total body weight over time was observed in the animals from control and different treatment groups (Supplementary File 1, Fig. S4).

Immunohistochemical assessment of formalin-fixed tumor tissues showed that the expression of ki67 proliferation marker was markedly decreased in all three treatment groups compared with the vehicle-treated controls, with the highest reduction in the SAM + 25(OH)D cohort (Fig. 2e). This further validates the reduction of tumor volumes seen in the animals receiving combination therapy at the protein level.

Virgin female MMTV-PyMT mice spontaneously develop lung metastases that arise from the primary breast tumor by 10–12 weeks (70–84 days) of age,^24^ which allows assessing the antimetastatic potential of a treatment regimen. On day 77, animals from all four groups were sacrificed, and lung tissues were collected. The extent of visceral metastasis mediated by the breast tumor cells was assessed by evaluating the formalin-fixed paraffin-embedded sections of the entire lung tissue. We found that breast tumor cells invaded the lung in all four groups (Fig. 2f, left panel). However, the area of lung metastases showed a significant decrease in the treatment groups compared with the vehicle-treated control animals, as determined by the measurement of the total area of micrometastases in the lungs (Fig. 2f, right panel). Moreover, the SAM + 25(OH)D combination-treated animals showed the lowest metastatic burden amongst the three treatment groups. These results suggest that SAM + 25(OH)D combination treatment cannot block the development of lung metastases but significantly reduces them.

The efficacy of a therapeutic molecule is dependent on its serum bioavailability for a reasonable period of time that will allow its absorption and subsequent distribution to the target tissues.^8^ We, therefore, performed a time-course experiment to determine the duration of SAM bioavailability in the serum following oral administration using LC-MS/MS. We found that SAM reaches its peak 30 min after administration, and its level drops down to the baseline after 240 min suggesting possible uptake by different tissues (Supplementary File 1, Fig. S5a). Next, we compared the SAM levels in the control and SAM-treated experimental MMTV-PyMT animals at sacrifice on week 11 and found a 3.6 fold increase in SAM concentration in the experimental group (Supplementary File 1, Fig. S5b).

We then checked the serum bioavailability of the intraperitoneally injected 25(OH)D by LC-MS/MS and found a significant elevation of the metabolite in the treated animals compared with the controls (Supplementary File 1, Fig. S6a). In addition, 25(OH)D injection elevated the levels of 1,25(OH)_2_D and 24,25(OH)_2_D in the serum of the experimental animals (Supplementary File 1, Fig. S6b, c). Taken together, these results suggest both SAM and 25(OH)D are bioavailable at the doses used in this study.

### Combination of SAM + 25(OH)D represses breast tumor cell growth in the skeleton in a syngeneic intratibial mouse model

**Fig. 3:**
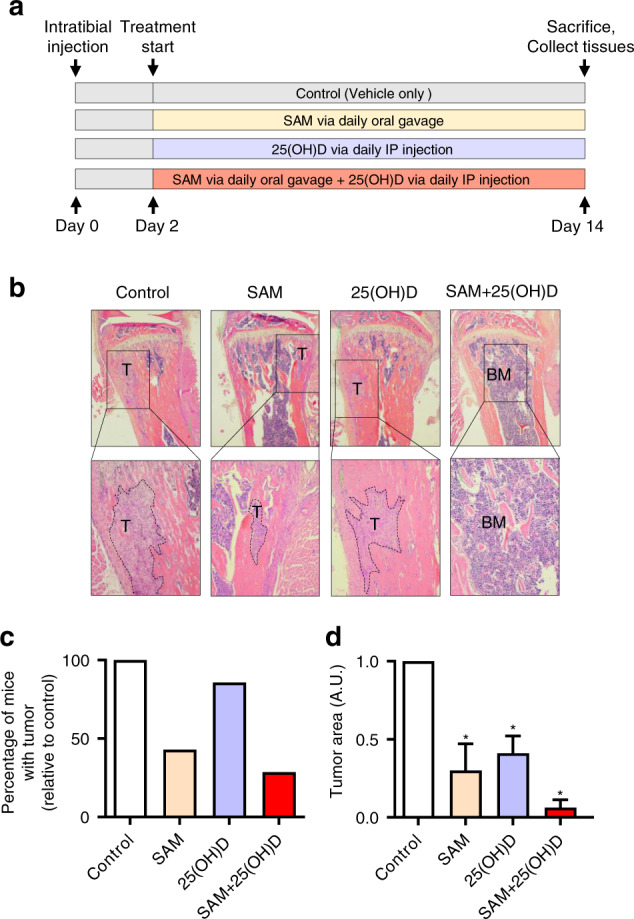
Effect of SAM, 25(OH)D, and their combination on breast cancer cell colonization to the bone in an intratibial model. **a** Schematic representation of treatment protocol used for treating female mice (FVB background) injected with PyMT-R221A cells and then treated with vehicle alone as control, SAM (160.0 mg·kg^−1^ per day) by daily oral gavage, 25(OH)D (40.0 ng·kg^−1^ per day) by intraperitoneal (i.p) injection, and SAM + 25(OH)D in combination from day 2 until day 14 when the mice were sacrificed (*n* = 9 animals per group). The tibias were collected from all animals and fixed for further histologic studies. **b** Representative low (×40; upper panel) and high (×100; lower panel) magnificaion images of the formalin-fixed histologic sections of the decalcified tibias from control and treated animals stained with Haemotoxylin and Eosin (H&E), where the tumors are marked a ‘T’ and bone marrow as ‘BM’. **c** Bar graph representing the percentage of mice that developed skeletal tumors in each group relative to the control group. **d** The relative area of tumor growth was quantified using the Fiji plugin (ImageJ), and the results are shown as mean ± SEM (*n* = 9 per group). Significant differences were determined using ANOVA followed by *post hoc* Tukey’s test and are represented by asterisks

We then assessed the effect of SAM + 25(OH)D in reducing the establishment of breast tumors on the skeleton using an immunocompetent syngeneic intratibial mouse model. The PyMT-R221A cells were utilized for intratibial implantation into female FVB mice as they were originally extracted from MMTV-PyMT mammary tumors,^16^ and also to keep a consistency of genetic background between the different in vivo models used in the study. Following a treatment strategy shown in Fig. 3a, animals from control and different treatment groups were treated daily from day 3 post tumor cell injection until sacrifice on day 14. Previous studies have demonstrated that by this time point, the cortical bone becomes compromised by these tumor cells, and they start to grow in the soft tissues in the surrounding areas.^25,26^ After sacrifice, tibias from all animals were collected, and H&E staining of the fixed paraffin-embedded bone tissue sections revealed that the percentage of animals that developed tumors was lower in the combination-treated group than that of control as well as the monotherapy treated groups (Fig. 3b, c). In addition, the skeletal tumor area was the smallest in the combination arm relative to monotherapy treated animals (Fig. 3d). Collectively, these observations suggest that the SAM + 25(OH)D combination treatment can reduce breast tumor growth in the skeleton, which is a major site where breast tumor cells migrate and establish to cause a secondary tumor in clinical settings.

### Effect of the combination treatment on PyMT-R221A transcriptome

**Fig. 4:**
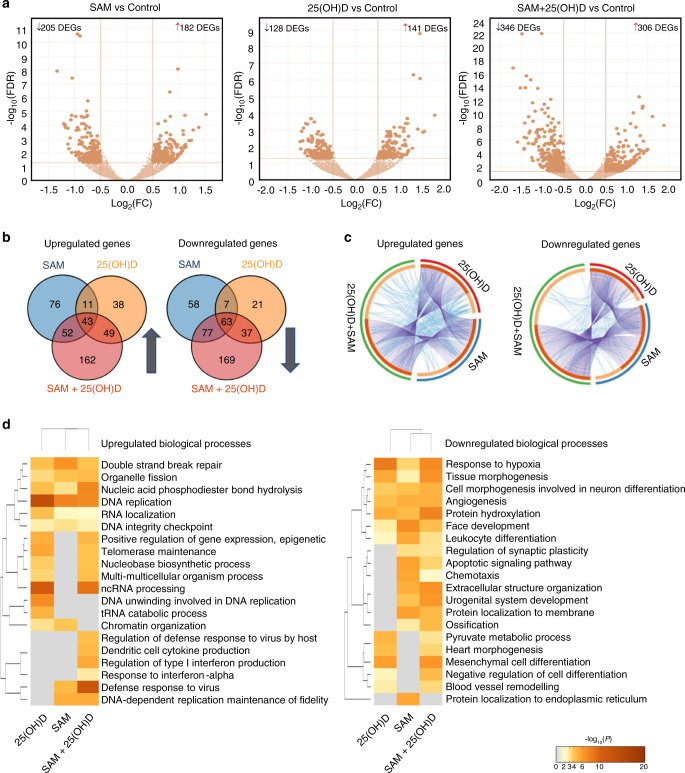
Transcriptome analyses of PyMT-R221A cells. Murine PyMT-R221A cells were treated with vehicle (control), 200 μmol·L^−1^ SAM, 100 nmol·L^−1^ 25(OH)D and a combination of SAM and 25(OH)D every other day for three times (on days 0, 2, 4) using the in vitro treatment protocol described in Fig. 1a. At the end of the experiment, RNA extracted from control and different treatment groups were subjected to RNA-Seq analyses. **a** The volcano plots of the significantly differentially expressed genes are shown [log_10_(FDR) versus log_2_FC]. **b** Venn diagrams representing the frequency of common and unique genes among different treatment groups. **c** Circos plot further representing the commonality and uniqueness of the functionalities of the up- and downregulated genes from each group. Each gene from the up- or downregulated gene lists has a spot on the arc. The dark orange color represents genes that are present in multiple treatment groups, and the light orange color shows genes that are unique for a treatment. In addition, the purple lines indicate the common genes that are found in different treatment groups, and the blue lines represent genes that are different but have similar functions. **d** Comparative heatmap of the Gene Ontology (GO) enrichment for biological processes for the different treatment groups, as determined by Metascape^52^

To characterize the molecular mechanisms underpinning the enhanced anticancer effect of the combination versus the effects of the monotherapy treatments with either SAM or 25(OH)D, we compared the drug-induced changes of PyMT-R221A transcriptome by RNA-Sequencing (RNA-Seq) of control (vehicle), 200 µmol·L^−1^ SAM, 100 nmol·L^−1^ 25(OH)D, and SAM + 25(OH)D treated samples. The differentially expressed genes (DEGs) between control and different treatment groups were delineated using DeSeq 2 (log_2_ fold change > 0.5 and FDR < 0.05). A total of 387 (182 upregulated and 205 downregulated), 269 (141 upregulated and 128 downregulated), and 652 (306 upregulated and 346 downregulated) DEGs were detected in SAM versus control, 25(OH)D versus control, and SAM + 25(OH)D versus control groups, respectively (Fig. 4a, Supplementary File 2). Hierarchical clustering of the top 50 DEGs in the three groups is shown separately in Supplementary File 1, Fig. S7. The number of common and unique genes that are differentially up- and downregulated in different treatment groups illustrated by the Venn diagrams showed that the transcriptomic footprint of the combination therapy is broader than any of the single treatments (Fig. 4b). Circos plots revealed the numerical and functional overlaps between the up- and downregulated genes from the different treatment groups (Fig. 4c). The combination treatment provides unique functionality compared with the single-agent treatment as shown by the higher number of blue lines within the Circos plots of up- and downregulated genes in combination treatment compared with the single-drug treatment. Interestingly, SAM, 25(OH)D and SAM + 25(OH)D treatments commonly target 106 genes (43 upregulated and 63 downregulated) suggesting overlap in molecular targets of these agents (Fig. 4b, Supplementary File 1, Fig. S8). Importantly, the combination treatment was not just a summation of the two monotherapies, but it had its unique footprint which involved changed expression of 331 genes (162 upregulated and 169 downregulated) indicating the possible modulation of additional biological signaling pathways.

A comparative Gene ontology (GO) enrichment analyses using the DEGs from all three treatment groups revealed that the genes upregulated by SAM + 25(OH)D are involved in crucial biological processes like regulation of type I interferon production (GO: 0032479), defense response to virus (GO: 0051607), while significantly downregulated genes by the combination are involved in key cancer-related processes such as response to hypoxia (GO:001666), angiogenesis (GO:0001525), and others as listed in Fig. 4d.

### Validation of the DEGs from the top enriched signaling pathways affected by the combination treatment

**Fig. 5:**
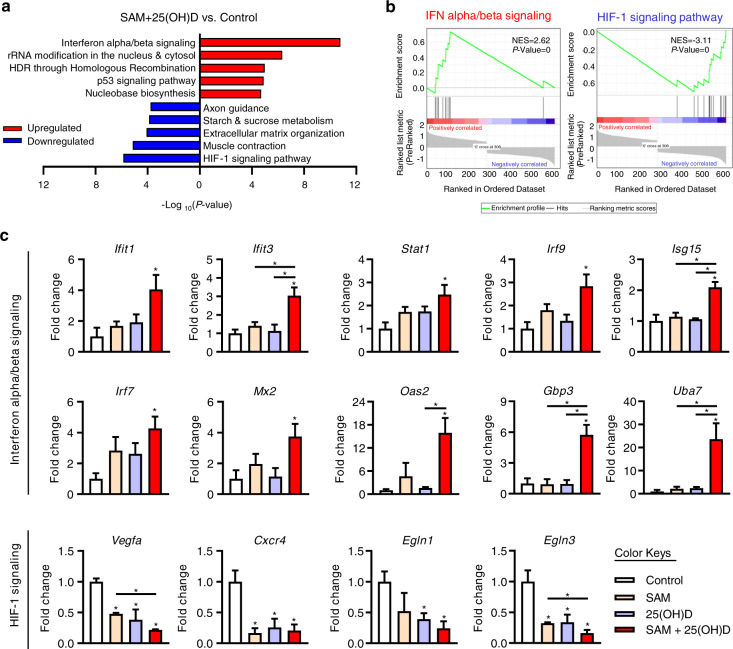
Functional validation of the genes identified by RNA-Seq. **a** Pathway analysis of the up- and downregulated DEGs from the SAM + 25(OH)D combination-treated group was done by using the KEGG and Reactome databases. Top five up- and downregulated pathways are shown as bar graphs. **b** GSEA analyses further showed the enrichment of the genes from ‘interferon alpha/beta signaling’ and ‘HIF-1 signaling pathway’. **c** RNA obtained from the control and treated PyMT-R221A cells was subjected to qPCR to validate the expression of selected genes from the ‘interferon alpha/beta signaling’ and ‘HIF-1 signaling' pathways. Results are shown as mean ± SEM of samples obtained from at least three different experiments per group. Significant differences are represented by asterisks

To gain further insight into the functional pathways that were significantly affected by the SAM + 25(OH)D combination treatment, a pathway enrichment analysis was done using the Kyoto Encyclopedia of Genes and Genomes (KEGG) and Reactome databases (Fig. 5a). We found that the top pathway significantly enriched by genes upregulated in response to the combination treatment is the ‘interferon alpha/beta signaling’ pathway (Fig. 5a). On the other hand, the top signaling pathway enriched for genes downregulated by the combination treatment is the ‘HIF-1 signaling pathway’ (Fig. 5a). Gene Set Enrichment Analysis (GSEA) further confirmed the enrichment of genes from the interferon alpha/beta and HIF-1 signaling pathways upon combination treatment (Fig. 5b). We also performed pathway analysis of the 106 common DEGs shared by SAM, 25(OH)D, and SAM + 25(OH)D combination treatment and found that the ‘HIF-1 signaling pathway’ is the top signaling pathway enriched by these genes (*P* = 3.42 × 10^−07^; Supplementary File 1, Fig. S9). We then analyzed the 331 DEGs that are uniquely regulated by the SAM + 25(OH)D combination treatment and found a significant enrichment of the ‘interferon alpha/beta signaling’ pathway (*P* = 3.33 × 10^−11^; Supplementary File 1, Fig. S10).

We next validated the RNA-Seq results on selected genes from the top upregulated (interferon alpha/beta signaling) and downregulated pathways (HIF-1 signaling pathway) using quantitative polymerase chain reaction (qPCR) analysis. Several crucial genes from the ‘interferon alpha/beta signaling’ pathways were significantly upregulated in the SAM + 25(OH)D combination-treated cells only but not by either of the monotherapies (Fig. 5c). On the other hand, in comparison with the control group, a significant decrease in the expression of selected genes from the ‘HIF-1 signaling pathway’ were observed in the combination-treated group, a trend that is also shared by the monotherapy treatments with either SAM or 25(OH)D (Fig. 5c). Similar results were also seen when tumoral RNA from control and treated animals (from Fig. 2) were analyzed by qPCR (Supplementary File 1, Fig. S11). We also measured the levels of *Vdr, Cyp27b1*, and *Cyp24a1* in control and treated PyMT-R221A cells and found their expression to be differentially regulated upon combination treatment relative to the control cells (Supplementary File 1, Fig. S12).

Since Stat1 plays a key role in mediating the immune responses activated by the interferon signaling pathway through the transcriptional regulation of several interferon related genes including *Irf7, Isg15*, *Oas2, Gbp3*—all of which are upregulated by the SAM + 25(OH)D (Fig. 5c), we next tested the hypothesis that upregulation of Stat1 in part mediates the enhanced anti-cancer effect seen by the combination treatment. For that, we compared the effect of Stat1 activator [2-(1,8-naphthyridin-2-yl)-Phenol; in short ‘2-NP’] and SAM + 25(OH)D treatment on PyMT-R221A cell proliferation. Treatment with either 2-NP or SAM + 25(OH)D both reduced tumor cell proliferation (Supplementary File 1, Fig. S13a). Interestingly, a triple combination of 2-NP with SAM + 25(OH)D showed an additive effect in reducing proliferation (Supplementary File 1, Fig. S13a). The expression of *Irf7*, a known transcriptional target of Stat1, was found to be elevated by either 2-NP or SAM + 25(OH)D treatment with a further elevation of its gene expression in the triple combination (Supplementary File 1, Fig. S13b). Taken together, these results indicate the possible involvement of the interferon signaling pathways in mediating the anticancer effects shown by the SAM + 25(OH)D combination treatment.

### Comparison of the DEGs upon combination treatment with publicly available breast cancer dataset

**Fig. 6:**
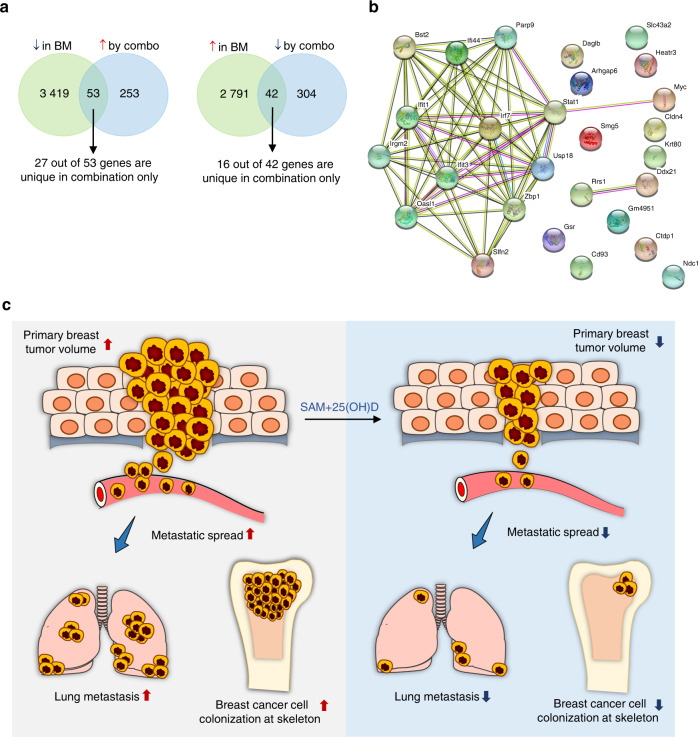
Comparison of DEGs in response to SAM + 25(OH)D with the DEGs in murine bone metastasis dataset. **a** Venn diagram of the up- and downregulated genes following combination treatment with SAM + 25(OH)D that overlapped with differentially expressed genes in a murine model of breast cancer bone metastasis in GSE37975. Here, BM: Bone metastasis **b** Molecular interaction networks of the encoded proteins from the 27 uniquely upregulated genes in the combination treatment are determined by the STRING database. **c** Summary of the anticancer effects mediated by SAM + 25(OH)D in vitro and in vivo. The combination treatment causes a significant reduction in cell proliferation and colony formation in a battery of breast cancer cell lines in vitro and reduces primary mammary tumor volume, visceral, and skeletal colonization by breast tumor cells in vivo. These anticancer effects were mediated through the differential regulation of key cancer and immune-related pathways, as shown by the transcriptome analyses

We compared the DEGs in response to SAM + 25(OH)D combination treatment from our study with genes that are differentially regulated in a publicly available dataset of mouse model of spontaneous bone metastasis determined by Affymetrix mouse 430 v2.0 gene expression arrays^27^ (GSE37975). Differentially expressed transcripts from GSE37975 were analyzed using the GEO2R tool from the NCBI GEO website and a total of 6 305 (2 833 upregulated and 3 472 downregulated) differentially expressed unique genes were obtained in the skeletal metastasis (spine) samples compared with the controls (at FDR <0.05). We then overlapped 2 833 upregulated and 3 472 downregulated genes from GSE37975 with the 346 downregulated and 306 upregulated genes upon SAM + 25(OH)D treatment from the current study. Our analysis showed that 53 transcripts that are downregulated in metastatic bone tissues, according to GSE37975, significantly overlapped with genes upregulated by SAM + 25(OH)D treatment as compared with the vehicle-treated control PyMT-R221A cells (hypergeometric test, *P* ≤ 0.05) (Fig. 6a, Supplementary Fig. S14). More interestingly, out of these 53 transcripts, 27 were uniquely upregulated by the SAM + 25(OH)D combination. On the other hand, the overlap of 42 transcripts (16 hits unique for combination only) that are upregulated in the mouse model of bone metacstasis but downregulated by SAM + 25(OH)D combination was not statistically significant by hypergeometric test (Fig. 6a, Supplementary Fig. S15). Next, we focused on the 27 genes that are downregulated in bone metastasis but uniquely upregulated by the combination. Protein-protein interaction (PPI) network analysis revealed a significant enrichment (PPI enrichment *P* < 1.0e-16) with ‘response to virus’ and ‘type I interferon signaling pathway’ as the top two most significantly enriched GO-pathways within the network (Supplementary File 1, Fig. S15).

Next, we overlapped the DEGs responding to SAM + 25(OH)D treatment with the ortholog human breast cancer patient gene list obtained from the BioXpress database^28^ and we found a list of 87 genes (36 are unique in combination only) that were upregulated in human breast tumors but downregulated by the combination treatment while there were 59 genes that were downregulated in patients but are upregulated by the combination treatment (Supplementary File 1, Fig. S16). However, the overlap of genes did not reach statistical significance by a hypergeometric test. Nevertheless, pathway analysis of the overlapped genes revealed differential regulation of several known cancer-related pathways like HIF-1 and HIF-2 transcription factor networks and β3 integrin cell surface interactions (Supplementary File 1, Fig. S16).

## Discussion

It is now clear that the cancer phenotype involves concurrent alterations in multiple gene pathways where both hypermethylation of tumor-suppressor genes and hypomethylation of the tumor-promoting genes occur.^29–31^ We, therefore, combined the methylating agent SAM with another commonly used nutraceutical agent Vit. D, a compound acting via its nuclear receptor triggers epigenetic reprogramming as well as demethylation,^32,33^ so that both hypermethylation and hypomethylation meditated abnormalities of the cancer methylome can be targeted concurrently. We found that the combination treatment caused a significant delay in the time to forming spontaneous tumors in experimental transgenic mice as compared with vehicle-treated control MMTV-PyMT mice. In addition, the combination showed a markedly improved therapeutic effect in reducing tumor volumes compared with the controls as well as that of single-agent treatment. The combination also reduced the extent of the metastatic burden to the lung tissue, which is the major site where the primary tumor cells metastasize in this well-established model.^22^

Skeletal metastasis is one of the main complications associated with advanced breast cancer which leads to intractable bone pain, hypercalcemia, increased bone fragility, nerve compression resulting in high incidence of morbidity and mortality.^34,35^ Collectively, these complications are called skeletal-related events and significantly hamper the quality of life of cancer patients. Despite its widespread prevalence, only a few therapeutic options are available for skeletal metastasis.^36^ Moreover, most of the available therapeutic options are palliative and are directed towards relieving bone pain and reducing the destruction of bone tissue.^36^ Therefore, there is an unmet need for novel therapeutic intervention decreasing the secondary tumor growth to the skeleton. Towards these goals, we used the PyMT-R221A intratibial model of breast cancer colonization to the bone to assess the anti-cancer potential of SAM + 25(OH)D and found that the combination treatment significantly reduced tumor growth in the tibial region.

The dosage used for in vivo 25(OH)D administration was similar to the one described previously.^19^ However, instead of using an osmotic pump for 25(OH)D administration, we used intraperitoneal injection so that it can be clinically translatable to humans. This mode of delivery is advantageous as it avoids re-implantation of the osmotic pump every 4 weeks and assures a uniform delivery of 25(OH)D, which is not always possible to maintain via other means like a dietary supplementation. To test the efficacy of our treatment strategy, we measured the levels of 25(OH)D, 1,25(OH)_2_D and 24,25(OH)_2_D in the serum of control and 25(OH)D injected animals and found elevated levels of these metabolites that suggested bioavailability of 25(OH)D (Supplementary File 1, Fig. S6). Based on our previous studies and results from our preliminary studies in these models using different doses of SAM (data not shown), 160 mg·kg^−1^ per day via oral gavage was found to be most effective which was used in the current study. Using this dose, we have observed an elevated SAM concentration in the serum of experimental animals treated with SAM compared with the control animals (Supplementary File 1, Fig. S5b). SAM is available as an approved dietary supplement for depressive disorders. However, in at least two different clinical trials, treatment with SAM ironically showed some transient behavioral abnormalities in a small number of participants.^37,38^ To assess whether SAM elicits any potential behavioral adversities at the dose used in this study, we conducted an open field test. However, we did not observe any behavioral abnormalities in the SAM-treated animals when compared with the vehicle-treated control animals (Supplementary File 1, Fig. S17).

One of the major aspects of the current study was to assess and compare the transcriptomic changes induced by single agents and their combination to understand the molecular footprint of the combination treatment as a possible explanation for its anticancer activity. We, therefore, performed RNA-Seq analyses of samples obtained from control and all three treatments and compared their expression profiles. We chose to analyze the transcriptome of mouse PyMT-R221A cells so that the anticancer effects seen in vivo can be directly linked to the molecular changes seen in the cancer cells in vitro since these cells were initially isolated from the MMTV-PyMT tumor.^39^ We found that the combination treatment has a much broader footprint than either of the monotherapies alone, but it also shared 106 genes (43 upregulated, 63 downregulated) with both SAM and 25(OH)D monotherapy treated groups which account for 12.6% of the total DEGs in all three groups (Fig. 3c). GO analysis revealed that treatment with SAM + 25(OH)D might boost the immune system through modulation of immune-related genes and might be considered for enhancement of other immunotherapy regimens. Analysis of publicly available murine breast cancer bone metastasis datasets revealed that interferon regulatory factor *Irf7*, whose expression is repressed during bone metastasis (GSE37975), is upregulated by the SAM + 25(OH)D combination treatment. It has been shown that *Irf7* repression promotes bone metastasis through immune escape in a mouse model of breast cancer bone metastasis.^27^ Moreover, overexpression of IRF7 inhibited prostate cancer cell-mediated bone metastasis in mice,^40^ suggesting a common role of the Irf7 axis in bone metastasis mediated by different types of malignancies.

Our molecular analysis of the effect of the combination therapy on the transcriptome shows that the combination regulates a new molecular landscape than just a sum of both monotherapies explaining the expanded anti-cancer activity of the combination. The combination of SAM + 25(OH)D targets important biological processes for cancer that would not be hit using either monotherapies (Fig. 4d). The 331 genes (162 upregulated, 169 downregulated) unique for the combination-treated groups target a wide array of pathways (Supplementary File 1, Fig. S10), of which the most notable are the immune-related ones (for example, response to type I interferon). The upregulation of the immune-related genes upon the combination treatment might induce an anti-viral immune response against the cancer cells, which could not be possibly attained at the same extent by either of the monotherapies. Moreover, these immune-related genes not only elicit better antitumor effects^41^ but also provide a better antimetastatic response in the bone microenvironment.^27,40^ This might be a possible reason behind the enhanced antitumor and antimetastatic response seen in animals receiving SAM + 25(OH)D combination in both transgenic and intratibial models of breast cancer. However, further experimental evidence is needed to confirm the exact role of the immune system in mediating these anticancer effects. We also found that several noncoding RNAs are changed upon SAM + 25(OH)D combination treatment, of which the most notable is the downregulation of the known oncogenic long non-coding RNA called *Rmrp* (Supplementary File 1, Fig. S18). This implies that the molecular changes mediated by the combination are not only limited to genes with coding potential, but those with no known peptides are also regulated by SAM + 25(OH)D, which warrants future in-depth investigation of the non-coding repertoire of the transcriptome. Moreover, SAM as a methylating agent may have a profound impact on chromatin accessibility through its ability to methylate DNA and histone proteins, which in turn might be responsible for many of the gene expression changes seen in the RNA-Seq experiment. Future studies investigating the PyMT methylome and cistrome using the recently described methyl-ATAC-sequencing,^42^ as well as chromatin immunoprecipitation sequencing using antibodies against different histone modifications, may provide further mechanistic insights to the epigenomic changes induced by SAM treatment.

In summary, this study strongly demonstrates the anti-cancer therapeutic effect of SAM + 25(OH)D combination in breast cancer in vitro and in vivo (Fig. 6c), that may be used separately or in parallel with the current first-line therapy to improve patient outcomes. What is particularly attractive about this combination [SAM + 25(OH)D] is that both of them have a long safety record that positions them well for long term use as chemopreventive and therapeutic agents to reduce cancer-associated morbidity and mortality.

## Materials and methods

### Cell culture and treatments

Human MDA-MB-231 (ATCC^®^ HTB- 26^™^) and ZR-75-1 (ATCC^®^ CRL-1500^™^) cell lines were obtained from the American Type Culture Collection (ATCC; Manassas, Virginia). The mouse breast cancer PyMT-R221A and E0771 cell lines were kindly gifted by Dr. Conor C. Lynch (H. Lee Moffitt Cancer Center and Research Institute, Tampa, FL, USA) and Dr. Jean S. Marshall (Dalhousie University, Halifax, Nova Scotia, Canada) respectively. MDA-MB-231, PyMT-R221A, and E0771 cells were maintained in Dulbecco’s modified Eagle’s medium (DMEM) supplemented with 10% fetal bovine serum (FBS), 2 mmol·L^−1^ L-glutamine and 100 U·mL^−1^ penicillin-streptomycin sulfate. The ZR-75-1 cells were supplemented with RPMI-1640 containing 10% FBS, 2 mmol·L^−1^ L-glutamine and 100 U·mL^−1^ penicillin-streptomycin sulfate. The HBEC were obtained from Celprogen (Cat# 36056-01) and supplemented with manufacturer recommended human breast epithelial cell culture serum-free media (Cat# M36056-01).

Our recent studies have demonstrated that treatment with 200 μmol·L^−1^ SAM elicits anticancer effects in different cancer cells.^8^ Therefore, we used this dose for in vitro studies with all four cell lines. A human-grade SAM (Life Science Laboratories, Lakewood, NJ, USA) was used for all in vitro and in vivo experiments. On the other hand, 100 nmol·L^−1^ 25(OH)D (Cayman chemical company, Cat#9000683) was used in vitro as previously shown by others.^13,18^

### Cell proliferation and viability assay

MDA-MB-231, ZR-75-1, E0771, and PyMT-R221A breast cancer cells were plated onto 6-well plates containing 10% FBS supplemented growth media. The next day the cells were serum-starved for 24 h before treatment with 200 μmol·L^−1^ SAM, 100 nmol·L^−1^ 25(OH)D, a combination of SAM + 25(OH)D, and vehicle (ethanol) by direct addition to 5% charcoal-stripped FBS containing growth medium. Treatment was done every other day three times, and the culture media was replenished at the time of each treatment, as shown in Fig. 1a. At the end of the treatment period, the cells were trypsinized and counted using a Coulter counter (Model ZF; Coulter Electronics, Hertfordshire, UK). To determine whether these treatments show any effect on the viability of normal breast epithelial cells, a trypan blue cell viability assay was done. Briefly, HBEC cells were treated using the same protocol; however, there was no serum starving step as the culture media was already serum-free. At the end of the treatment period, the cells were trypsinized, washed with PBS, and then stained with 0.4% trypan blue (Sigma). The viable cells were counted directly using a light microscope.

### Clonogenic survival assay

After the completion of the usual in vitro treatment regimen, 5 000 cells from control and each treatment group were plated onto each well of standard six-well plates supplemented with FBS containing regular growth medium. The media was replenished every 3–4 days, and after 10–14 days from initial plating, the media was removed. The cells were then fixed with methanol: acetic acid at 3:1 ratio for 20 min at room temperature. Afterward, the fixing solution was removed, and the fixed cells were incubated for 15 min with the staining solution containing 0.5% crystal violet. The cells were then washed with water, dried overnight, and the next day the colonies were counted under a light microscope. A nonoverlapping group of at least 50 cells was considered as one colony, as described before.^8^

### In vivo models

All in vivo procedures were done in compliance with the McGill University Facility Animal Care Committee approved protocol. Two mouse models were used: MMTV-PyMT transgenic mice (FVB background) and syngeneic FVB mice (in which the PyMT-R221A cells were injected via intratibial route). Only the female animals were used for experiments, and the number of animals in one cage ranged between 2 and 5. All animals were housed at a 12 h light–dark cycle and had access to water and standard food (Teklad rodent diet 2918 containing 0.4% methionine and 1.5 IU·g^−1^ of Vitamin D3) ad libitum.

### MMTV-PyMT transgenic mice

The MMTV-PyMT mice develop spontaneous mammary tumors at around 5–6 weeks (Days 35–42) after birth and lung metastases arise by 10–12 weeks (Days 70–84).^24^ Therefore, on week 4 (Day 28) after birth, the MMTV-PyMT mice were randomized and treated in four different groups: phosphate buffer saline (PBS) as the vehicle-treated controls, a group of animal receiving 160.0 mg·kg^−1^ per day of SAM via oral gavage, a group receiving 40.0 ng·kg^−1^ per day 25(OH)D by intraperitoneal injection (i.p.) injection, and at the last group receiving both SAM and 25(OH)D (*n* = 8 per group). The diameters of primary mammary tumors were measured at weekly intervals using a caliper, and tumor volumes from different animals were calculated using the following formula: V = (length × Width^2^)/2. At the experimental endpoint, the animals were sacrificed, and different tissues were collected for downstream analysis. Tumor growth inhibition (TGI) at sacrifice was determined using the following formula: 100*(1 − T_t_/T_0_), where T_t_ and T_0_ refer to the average volumes of tumors for a given treatment group and control respectively.^43^

### Intratibial model for skeletal metastasis

Murine PyMT-R221A cells were implanted into the tibia of female syngeneic FVB mice to assess whether SAM, 25(OH)D, and SAM + 25(OH)D treatment could reduce breast tumor cell growth in the skeleton. The PyMT-R221A cells were initially isolated from MMTV-PyMT (or MMTV-PyVT) tumors^16^ and have been shown to form tumors within 2 weeks when injected into the intratibial region.^25,26^ Briefly, 2 × 10^5^ PyMT-R221A cells were injected into the tibial region of 4–6-week-old female FVB albino mice. On day 3 post-tumor cell implantation, the animals were randomized into four different groups and treated daily with vehicle (PBS), 160.0 mg·kg^−1^ per day of SAM via oral gavage, 40.0 ng·kg^−1^ per day 25(OH)D by i.p. injection and a combination of SAM + 25(OH)D until sacrifice on day 14 (*n* = 9 per group). Afterwards the tibias were collected, fixed using Periodate-Lysine-Paraformaldehyde (PLP) solution, and decalcified for further histological assessment. The decalcified tibias were then dehydrated and embedded in paraffin before Haemotoxylin and Eosin (H&E) staining at the Research Institute of the McGill University Health Centre (RI-MUHC) histopathology platform. Tumor area from the H&E stained bone sections was determined using the Image J (Fiji plugin) software.

### Immunohistochemistry

Immunohistochemical assessment of the formalin-fixed mammary tumor tissues from control and different treatment groups was done using an antibody against Ki67 (Cat# M7240, Dako, Glostrup, Denmark). The Ki67 positive proliferating cells from randomly selected fields from each group was determined by an automated approach using ‘ImmunoRatio’.^44^

### Measurement of serum levels of SAM, 25(OH)D, 1,25(OH)_2_D, and 24,25(OH)_2_D

For the time-course experiment of SAM bioavailability (Supplementary File 1, Fig. S5a), animals were treated with SAM and blood was collected by cardiac puncture at different time points (15, 30, 60, 120, and 240 min) after administration. We also collected blood from an animal just before administration and plotted it as the baseline (*t* = 0). The serum was collected from the supernatants after centrifugation. For the experimental animals (Supplementary File 1, Fig. S5b), serum was collected from 11-week-old SAM-treated mice within an hour after SAM administration by gavage. For comparison, serum was collected from the 11-week-old animals from the control group. Afterward, the protein contents of the serum were removed by acetonitrile precipitation, and the remainder was injected into the AB SCIEX SelexION^™^ (Framingham, Massachusetts, USA) for LC/MS-MS separation at the Proteomics Core Facility of the RI-MUHC. The data obtained were analyzed using Analyst TF 1.7 software (SCIEX, Framingham, Massachusetts, USA).

Measurement of serum 25(OH)D, 1,25(OH)_2_D, and 24,25(OH)_2_D levels in control and 25(OH)D treated animals was done by LC/MS-MS at the Heartland Assays Inc. (Ames, IA, USA).

### RNA extraction and quantitative real-time PCR (qPCR)

Total RNA was extracted using the AllPrep DNA/RNA Mini Kit (Qiagen; Cat# 80204) following the standard protocol provided by the manufacturer. The qPCR assay was performed following our previously described protocol.^12^ The list of primers used in this study is shown in Supplementary File 1, Tables S2 and S3. Gene expression changes between the control and different treatment groups were carried out as described previously.^45^

### RNA sequencing (RNA-Seq) and analysis pipeline

For RNA Seq, biological replicates from the vehicle (control), 200 μmol·L^−1^ SAM, 100 nmol·L^−1^ 25(OH)D, and SAM + 25(OH)D-treated PyMT-R221A cells was used (*n* = 3 for all except 25(OH)D where *n* = 2). Sample quality control was performed using the Agilent 2100 Bioanalyzer. Qualifying samples were then prepped following the standard protocol for the NEBnext Ultra ii Stranded mRNA (New England Biolabs). Sequencing was performed on the Illumina NextSeq 500 with paired-end 43 bp × 43 bp reads. The RNA-seq data were processed and interpreted with the Genialis visual informatics platform (https://www.genialis.com). An automated data analysis pipeline run in the Genilais platform consisted of the following: Sequence quality checks were performed on raw and trimmed reads with FastQC (http://www.bioinformatics.babraham.ac.uk/projects/fastqc). Trimmomatic was used to trim adapters and filter out poor quality reads.^46^ Trimmed reads were then mapped to the mouse (mm10) reference genome using the HISAT2 aligner.^47^ Gene expression levels were quantified with HTSeq-count,^48^ and differential gene expression analyses were performed with DESeq2.^49^ Lowly-expressed genes, which have expression count summed over all samples below 10, were filtered out from the differential expression analysis input matrix.

### Behavior test

The open field test was performed and analyzed as described before.^8^

### Statistical and bioinformatics analyses

Results are expressed as the mean ± standard error of the mean (SEM). Statistical significance was carried out by Student’s *t* test and ANOVA depending on the type of data. A *P* value of ≤0.05 was considered statistically significant. The statistical significance of the overlapping genes between different transcriptome-wide studies was determined by a hypergeometric test using RStudio, where the total number of genes was arbitrarily set at 25 000 to avoid cross-platform gene expression discrepancies. The pathway enrichment and GO analysis from different gene lists was carried out by using ConsensusPathDB,^50^ Gene Set Enrichment Analysis (GSEA),^51^ and Metascape.^52^ Protein-protein interaction was analyzed by the STRING database (https://string-db.org/).

## Supplementary information

Supplementary File 1

Supplementary File 2

## Data Availability

The authors declare that all relevant data generated or analyzed during this study are available within the main article and the supplementary files.
