# Supplementary material for: An enhanced chemopreventive effect of methyl donor S-adenosylmethionine in combination with 25-hydroxyvitamin D in blocking mammary tumor growth and metastasis
Source: Bone Res. 2020 Jul 22;8:28. doi: 10.1038/s41413-020-0103-6 (PMC7376160; doi:10.1038/s41413-020-0103-6)
Supplement: Supplementary file 1 [file 41413_2020_103_MOESM1_ESM.pdf]

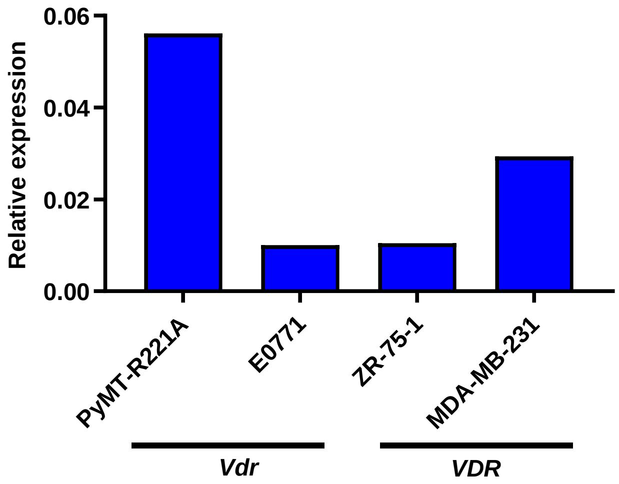

Figure S1: Expression of the gene encoding for vitamin D receptor in different human and murine breast cancer cell lines. RNA obtained from PyMT-R221A, E0771, ZR-75-1, and MDA-MB-231 cells were subjected to qPCR using mouse (*Vdr*) and human (*VDR*) specific primers to determine the basal levels of vitamin D receptor expression. The expression values were normalized using *Gapdh/GAPDH* as an internal loading control and expressed as a bar graph.

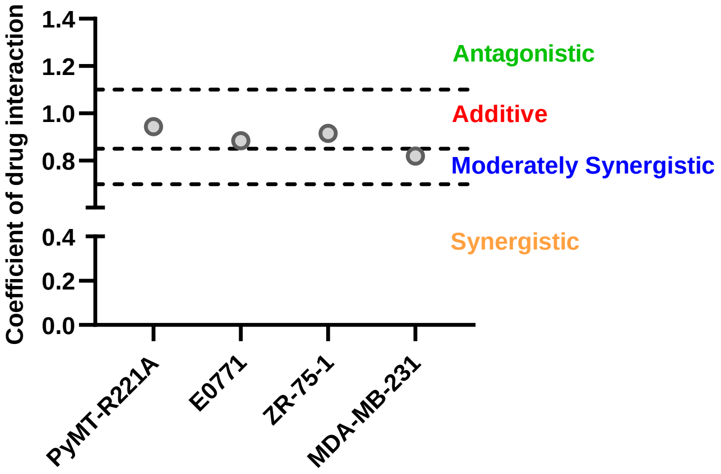

Figure S2: The coefficient of drug interaction (CDI) between SAM and 25(OH)D combination in different cell lines. The CDI values for each of the cell lines were calculated using the following equation, CDI = AB/(A × B). Here, AB: relative cell growth of the combination compared to control; A or B: relative cell growth of the single agent treated groups compared to the control. CDI < 0.7 indicates strong synergistic; CDI between 0.7 to 0.9 indicates moderately synergistic; CDI between 0.9 to 1.1 indicates additive and CDI > 1.1 indicates an antagonistic effect.

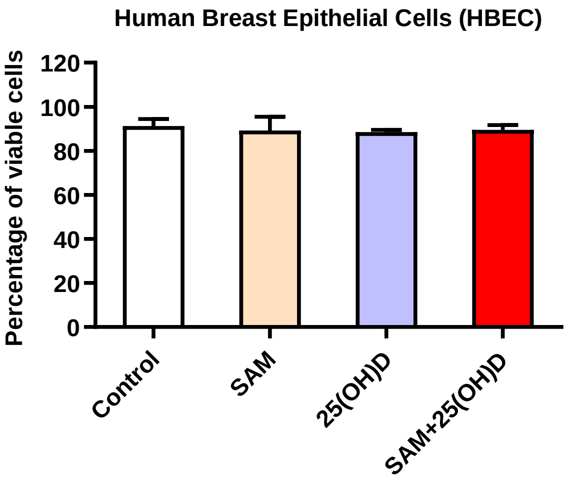

Figure S3: Effect on the viability of human breast epithelial cells (HBEC). HBEC cells were plated at the same density and treated with vehicle only control, 200 µM SAM, 100 nM 25(OH)D, and a combination of SAM+25(OH)D. At the end of the experiment, the cells were trypsinized, stained with trypan blue, and the total number of viable cells in each group was counted under a light microscope. Results are shown as the mean ± SEM (n=4). No significant difference was observed in the percentage of viable cells compared to the total number of cells in each group during the time of harvest, which indicated that the doses of the different anti-cancer agents used in this study are not toxic to the viability of the normal breast epithelial cells.

Table S1: Analyses of different biochemical parameters in the serum. The results are shown as mean ± SEM from three different mice in each group.

Parameter	Control	SAM	25(OH)D	SAM+25(OH)D
Calcium (mmol/L)	2.26 ± 0.05	2.26 ± 0.6	2.35 ± 0.02	2.40 ± 0.12
Total protein (g/L)	39.67 ± 1.36	42.33 ± 1.19	42.67 ± 0.27	44.67 ± 0.72
Albumin (g/L)	20.67 ± 0.54	22.33 ± 0.72	22.33 ± 0.27	21.67 ± 1.29
Albumin/Globulin ratio	1.1 ± 0.00	1.1 ± 0.00	1.1 ± 0.04	1.1 ± 0.04
Glucose (mmol/L)	16.1 ± 1.15	15.8 ± 0.49	15.5 ± 1.26	16.36 ± 0.66
BUN Urea (mmol/L)	9.23 ± 0.12	7.93 ± 0.48	7.23 ± 0.72	6.8 ± 0.23
Creatinine (µmol/L)	11 ± 0.81	10 ± 1.41	12.67 ± 0.54	11.33 ± 1.51
Total Bilirubin (µmol/L)	8.33 ± 1.09	9.33 ± 1.9	10.67 ± 0.98	8.67 ± 0.27
ALT (U/L)	56.6 7 ± 5.52	56.33 ± 3.95	41.67 ± 0.27	48.67 ± 3.81
Alkaline phosphatase (U/L)	71.5 ± 3.75	80.67 ± 13.37	76.67 ± 6.6	64.67 ± 4.65
Cholesterol (mmol/L)	2.82 ± 0.14	3.0 ± 0.11	2.79 ± 0.07	2.7 ± 0.18
Sodium (mmol/L)	143 ± 3.77	145.33 ± 3.81	152 ± 3.29	145.67 ± 6.36
Potassium (mmol/L)	4.16 ± 0.36	5.17 ± 0.14	4.8 ± 0.21	4.07 ± 0.22
Chloride (mmol/L)	110.67 ± 1.19	114 ± 2.05	118.67 ± 2.22	116.33 ± 4.72
Phosphorus (mmol/L)	2.79 ± 0.35	2.35 ± 0.03	2.64 ± 0.24	2.92 ± 0.13
Magnesium (mmol/L)	1.06 ± 0.03	1.0 ± 0.05	1.11 ± 0.06	1.05 ± 0.05

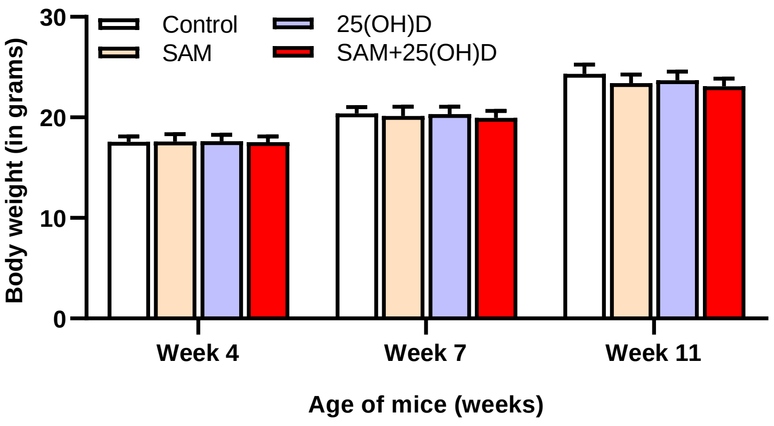

Figure S4: Total bodyweight of the transgenic MMTV-PyMT animals. The control and treated MMTV-PyMT animals are weighed at different time intervals from the beginning of the treatment regimen on week 4 after birth until sacrifice on week 11. No significant difference was observed in the total bodyweight of the animals from different treatment arms.

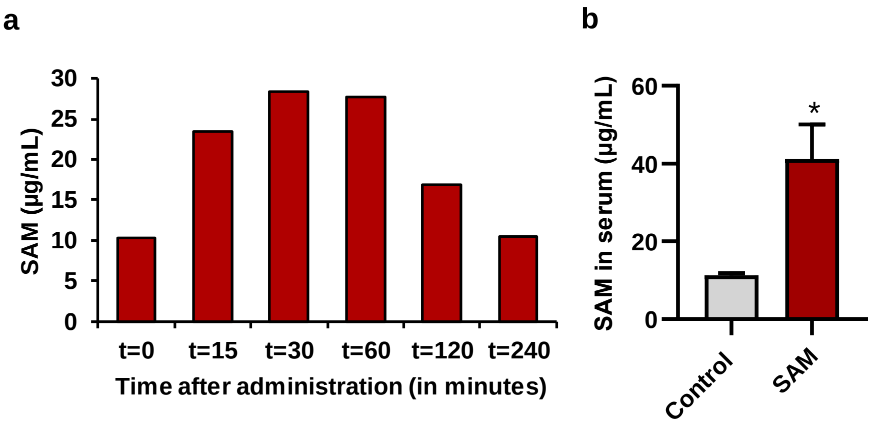

Figure S5: SAM bioavailability in the serum. a The SAM level in the serum of control and SAM-treated animals was assessed at different time points following oral gavage. Each bar represents the concentration obtained from the LC-MS/MS peak intensity for SAM at a specific time-point. Here, t=0 represents the baseline value of SAM just before treatment. The highest peak of SAM bioavailability in the serum was found between 30 to 60 minutes after administration, and the level comes down to the baseline at around 240 minutes after administration. **b** The serum from control (n=3) and SAM-treated (n=5) experimental animals were collected, and LC-MS/MS was done to determine the levels of SAM. Results are shown as the mean ± SEM. Significant differences were determined using a student's *t*-test and are represented by asterisks.

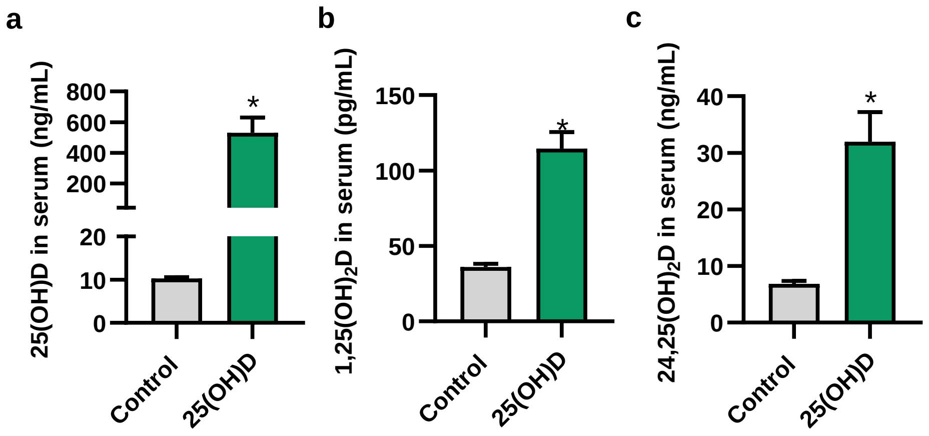

Figure S6: Serum levels of 25(OH)D,1,25$(OH)_2$D, and 24,25$(OH)_2$D. LC-MS/MS assays were done from the serum obtained from control (n=3) and 25(OH)D-treated (n=5) experimental animals to determine the levels of 25(OH)D (**a**), 1,25$(OH)_2$D (**b**) and 24,25$(OH)_2$D (**c**). Results are shown as the mean ± SEM. Significant differences were determined using a student's *t*-test and are represented by asterisks.

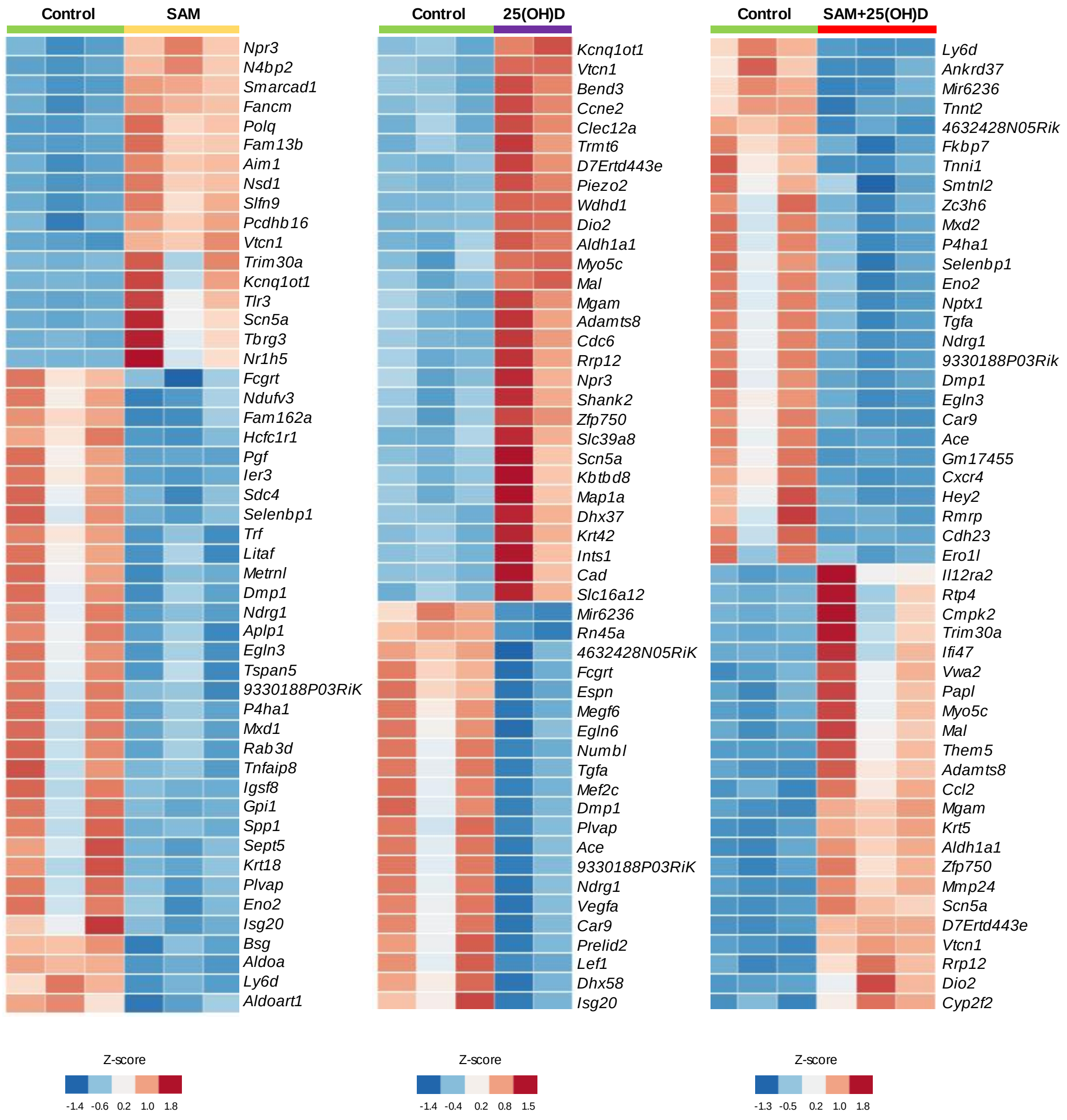

Figure S7: Heatmap of the top 50 significantly DEGs in each of the three treatment groups ($\log_2$ fold change>0.5 and FDR<0.05).

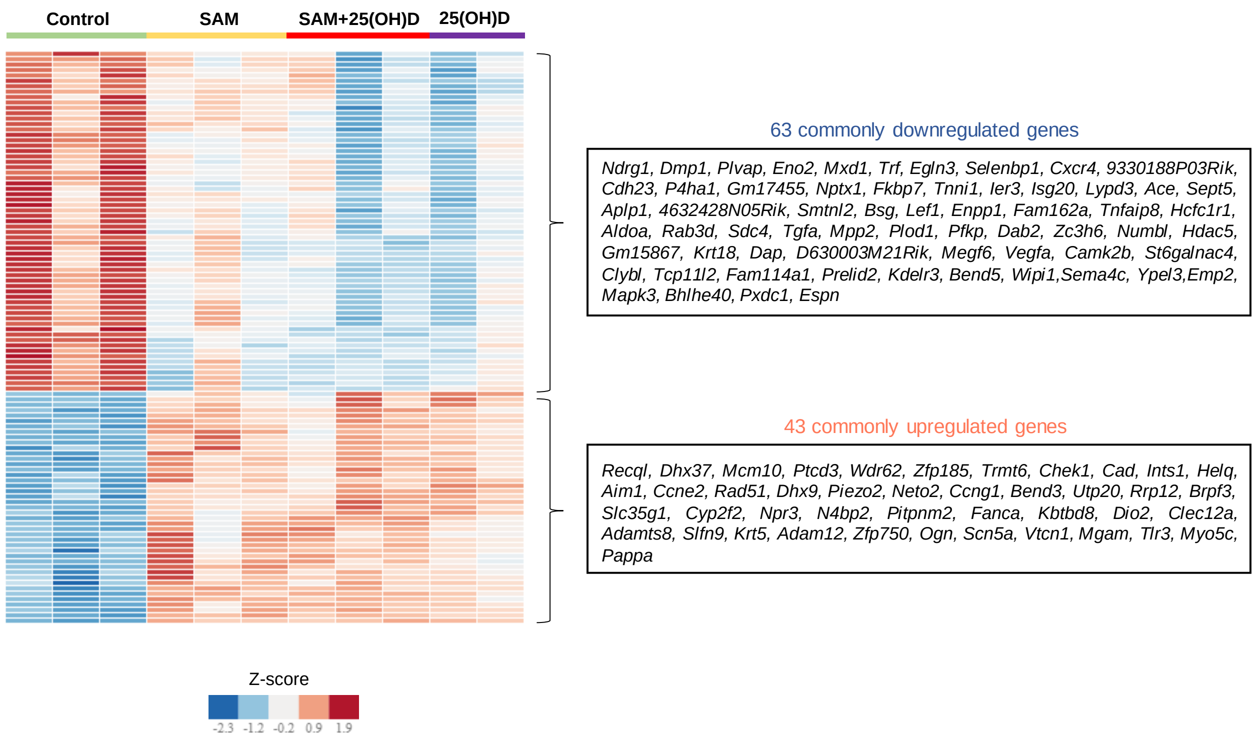

Figure S8: Heatmap of 106 common DEGs in each of the three treatment groups ($\log_2$ fold change>0.5 and FDR<0.05). The lists of the commonly up and downregulated genes are shown within the boxes on the right.

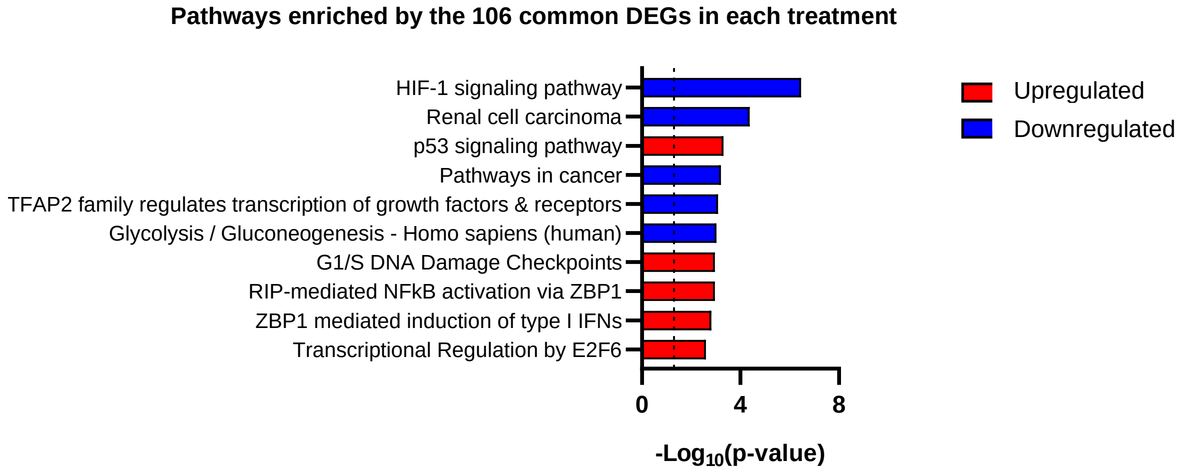

Figure S9: Enriched pathways affected by the genes that are commonly up and downregulated by all three treatment groups relative to the control PyMT-R221A cells. The pathways enriched with up and downregulated genes are shown in 'red' and 'blue', respectively. Top five up and top 5 downregulated pathways are shown together as a bar graph. The 'HIF-1 signaling pathway', which is enriched with the downregulated genes from all three treatment groups, showed the highest statistical significance (P= 3.42x10^{-07}).

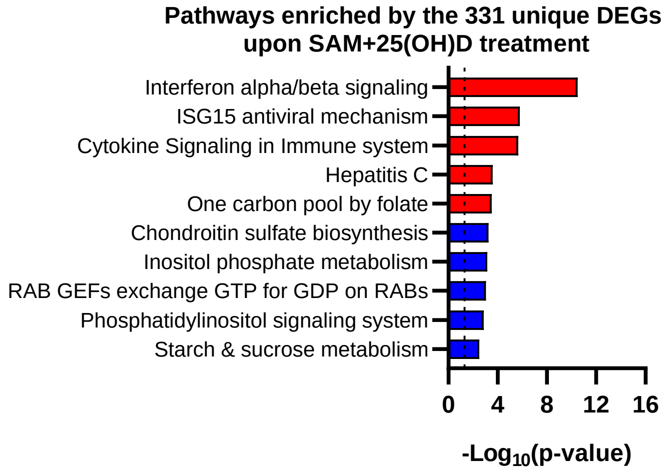

Figure S10: Enriched pathways and biological affected by the genes that are uniquely up and downregulated by SAM+25(OH)D treatment relative to the control PyMT-R221A cells. The pathways enriched with up and downregulated genes are shown in 'red' and 'blue', respectively. Top five up and top 5 downregulated pathways are shown together as a bar graph. An enrichment of several immune-related signaling pathways is seen when the analysis was done using the genes that are uniquely upregulated by SAM+25(OH)D. Interferon alpha/beta signaling pathway showed the highest statistical significance (P=3.33x10^{-11}).

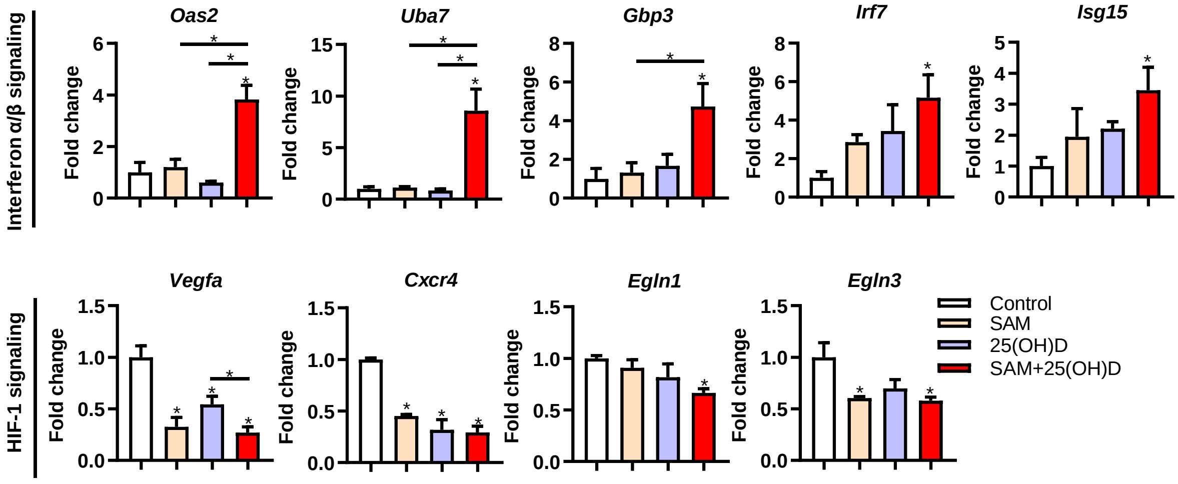

Figure S11: qPCR from RNA obtained from primary tumors. Briefly, total RNA obtained from the primary tumors of control and treated animals (from Figure 2) were subjected to qPCR to validate the expression of selected genes from the 'interferon alpha/beta signaling' and 'HIF-1 signaling pathway'. Results are shown as mean ± SEM of RNA obtained from at least three animals per group. Significant differences are denoted by an asterisk.

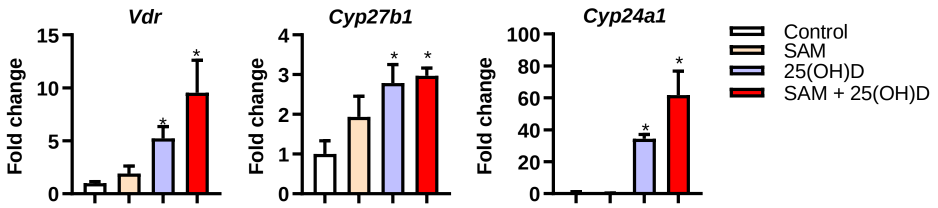

Figure S12: Expression of *Vdr, Cyp27b1,* and *Cyp24a1* in PyMT-R221A cells. Briefly, total RNA obtained from the control and treated PyMT-R221A cells were subjected to qPCR to validate the expression of *Vdr*, *Cyp27b1*, and *Cyp24a1*. Results are shown as mean ± SEM of RNA obtained from at least three different experiments. Significant differences are denoted by an asterisk.

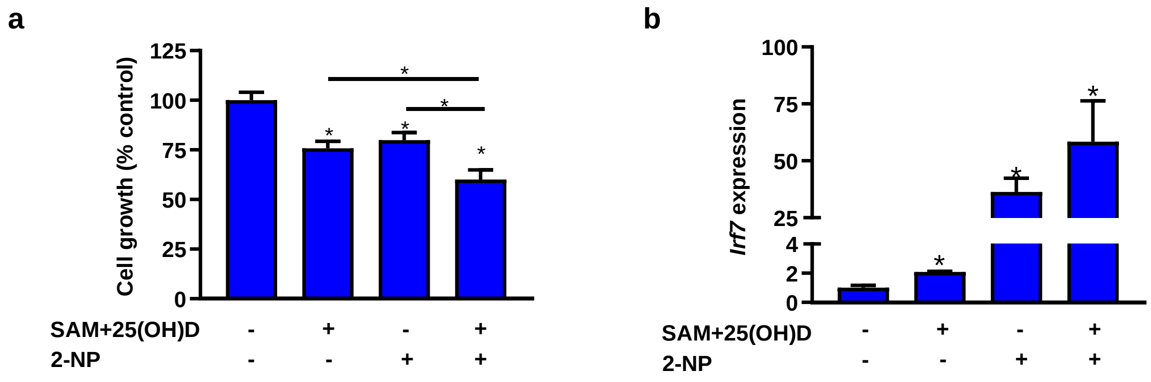

Figure S13: Effect of Stat1 activator on proliferation. **a** The PyMT-R221A cells were plated on 24 well plates and treated with vehicle control or SAM+25(OH)D for 24 hours. On the other hand, 25µM 2-(1,8-naphthyridin-2-yl)-Phenol (2-NP) [Sigma] was added directly into the media for 5 hours; the media was then removed and replenished with either regular culture media (for 2-NP monotherapy group) or media with SAM+25(OH)D for triple therapy treated group for 19 hours. At the end of 24 hours, cells were trypsinized and counted directed using a Coulter counter. Statistical analysis was done using ANOVA followed by *post hoc* Tukey's test from the data obtained from three independent experiments. **b** Total RNA obtained from the four different groups was subjected to qPCR to validate the increased expression of *Irf7*, which is a downstream target of the Stat1 transcription factor. A significant increase in the expression of *Irf7* was observed in all three treatment groups with the highest expression in the triple therapy group. It should be noted the cells were treated once for a period of 24 hours for this experiment in contrast to the other *in vitro* experiments during this study, where the cells were treated three times every second day. Hence, the level of *Irf7* increase upon SAM+25(OH)D treatment is less in Figure S13b compared to Figure 5c. Results are shown as mean ± SEM of RNA obtained from three experiments. Significant differences are denoted by an asterisk.

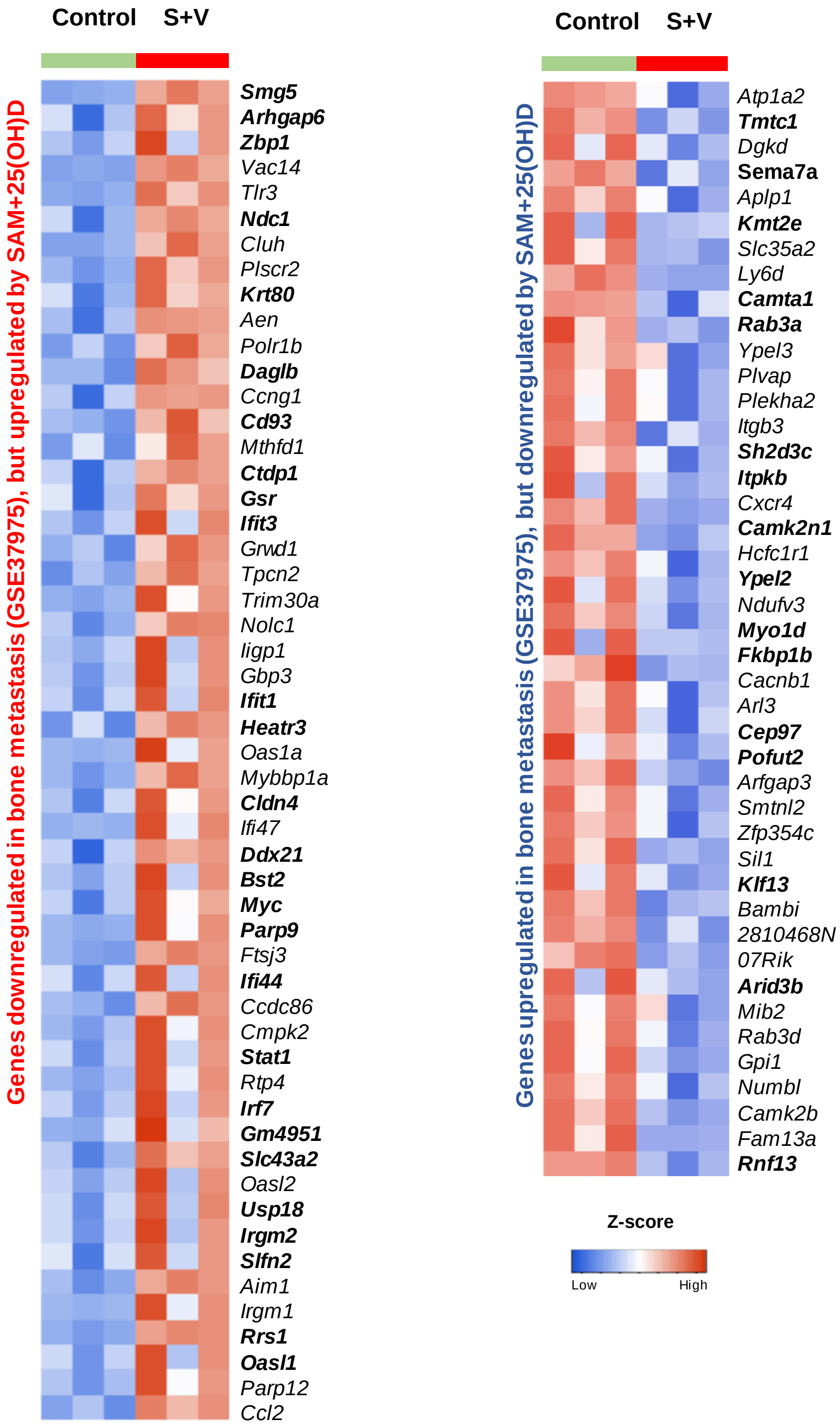

Figure S14: Heatmap of the significantly 53 upregulated and 42 downregulated genes following combination treatment with SAM+25(OH)D [denoted by 'S+V' in the figure], which are also differentially expressed in GSE76772 in the opposite direction. Moreover, 27 out of the 53 upregulated and 16 out of the 42 downregulated genes are significantly differentially regulated by the combination treatment only according to the cut-off set during RNA-Seq analysis. The genes unique in combination treatment only are shown by bold letters in the heatmap.

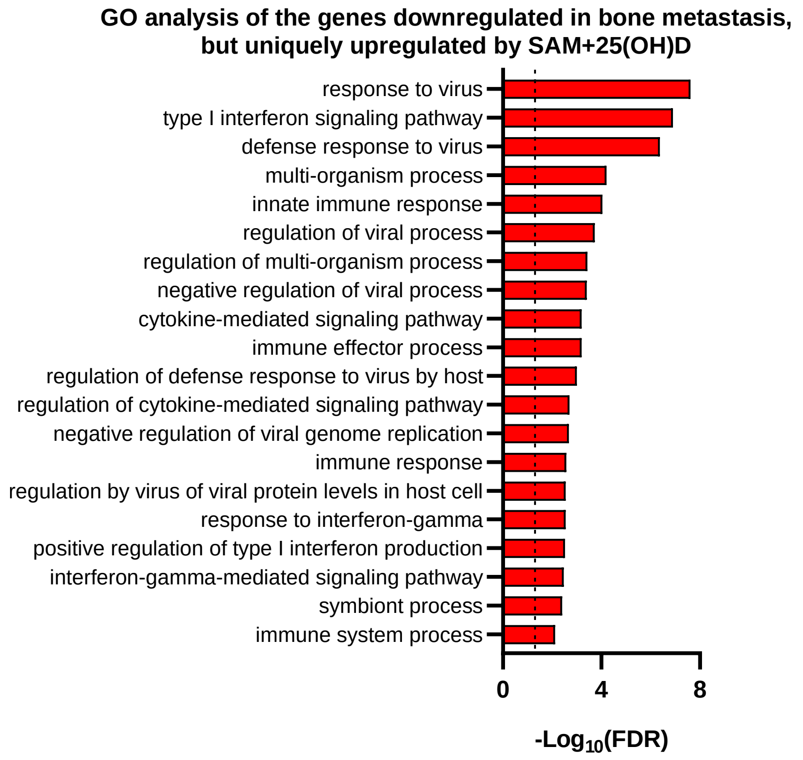

Figure S15: GO analysis of the 27 uniquely upregulated genes by SAM+25(OH)D that are downregulated in the bone metastasis dataset (GSE76772). The top 20 significant hits are shown as a bar graph where a significant enrichment of immune-related signaling pathways is seen.

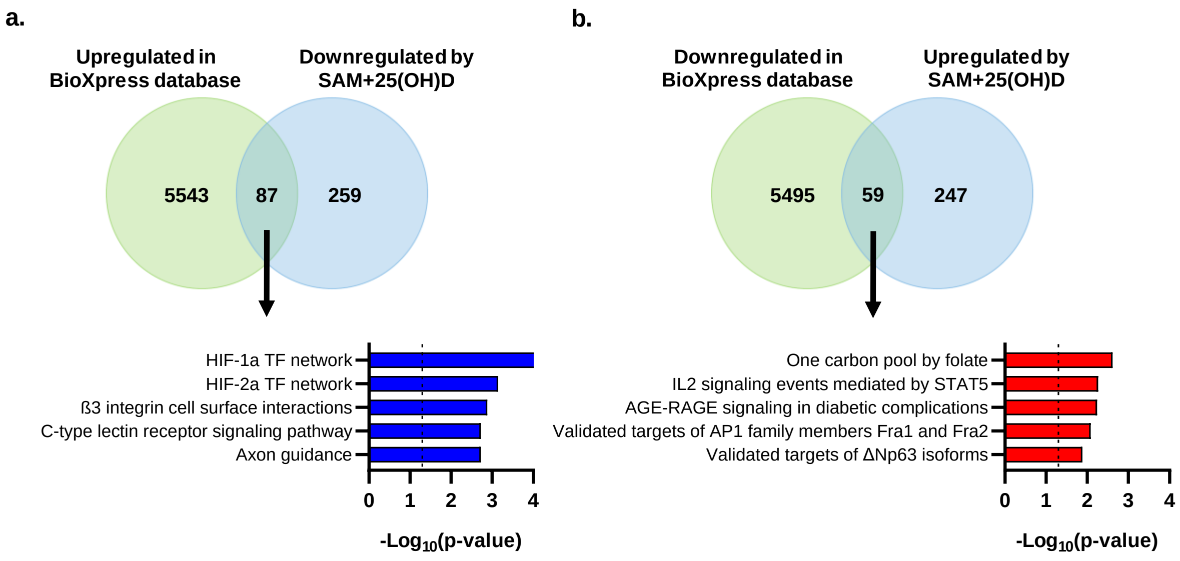

Figure S16: Comparison of DEGs in response to SAM + 25(OH)D with human breast cancer genes. **a** Venn diagram of the downregulated genes following combination treatment with SAM+25(OH)D showed an overlap of 87 genes that are upregulated in the human breast tumors in the BioXpress database. Pathway analysis of these genes is shown in the bar graph below (in blue). Out of these 87 overlapped genes, 36 are uniquely regulated by the combination only. **b** Venn diagram of the upregulated genes following combination treatment with SAM+25(OH)D showed an overlap of 59 genes that are downregulated in the human breast tumors in the BioXpress database. Pathway analysis of these genes is shown in the bar graph below (in red). Out of these 59 overlapped genes, 31 are uniquely regulated by the combination.

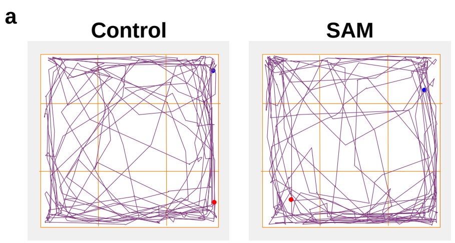

b

Open field test parameters	Control	SAM-treated	*P*-value
Total distance travelled (m)	29.7 ± 1.3	30.3 ± 1.9	0.83
Average speed (m/s)	0.1 ± 0.003	0.1 ± 0.006	0.74
Number of entries to the center zone	10.7 ± 0.9	10.6 ± 0.9	0.92
Time in the center zone (s)	7.5 ± 0.9	6.9 ± 0.9	0.65
Distance travelled in the center (m)	1.6 ± 0.1	1.6 ± 0.2	0.78

Figure S17: Assessment of animal behavior upon SAM-treatment. a Representative track plots from the video recordings of control and SAM-treated animals generated by using ANY-maze software are shown in the right panel. **b** Different parameters determined from the open field test of control and SAM-treated mice are provided in a tabular format. Results are shown as mean ± SEM (n=7). No significant alterations in animal behavior were observed upon SAM-treatment.

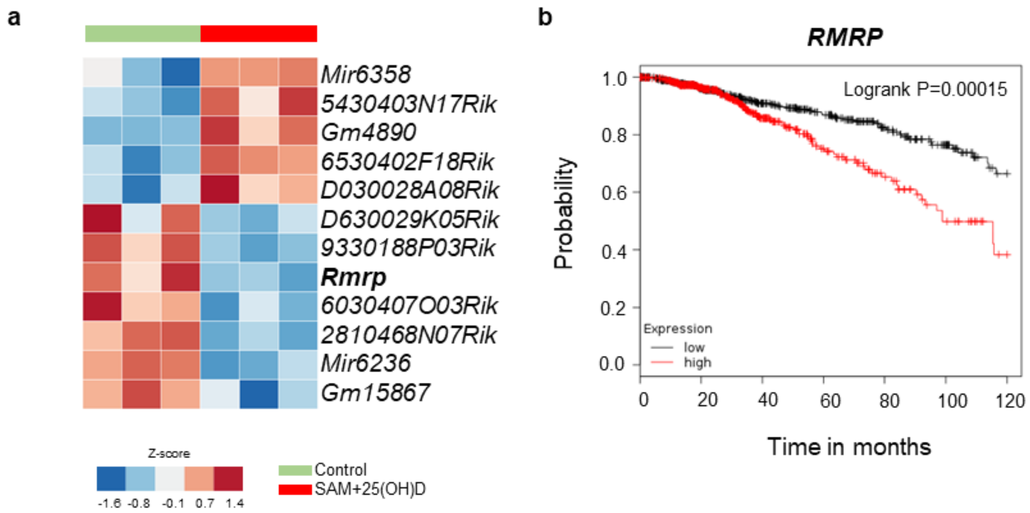

Figure S18: Differentially regulated non-coding RNAs upon SAM+25(OH)D combination treatment. a Heatmap of the significantly differentially expressed non-coding RNAs in SAM+25(OH)D combination relative to control cells. The expression of *Rmrp*, a known long non-coding RNA with oncogenic function, is downregulated by the combination treatment. **b** Kaplan-Meier survival plot from RNA-Seq data of 1089 breast cancer patients reveals that higher expression of the human *RMRP* gene is associated with poor overall survival.

Table S2: The mouse specific primers used in this study are listed below[1-9]

Gene Name		Sequences used for qPCR (5'→ 3')
Vegfa	For<br>Rev	CCACGTCAGAGAGCAACATCA<br>TCATTCTCTCTATGTGCTGGCTTT
Cxcr4	For<br>Rev	TCCTCCTGACTATACCTGACTTCATCT<br>CCTGTCATCCCCCTGACTGAT
Egln1	For<br>Rev	GCCCAGTTTGCTGACATTGAAC<br>CCCTCACACCTTTCTCACCTGTTAG
Egln3	For<br>Rev	AGGCAATGGTGGCTTGCTATC<br>GCGTCCCAATTCTTATTCAGGT
Irf7	For<br>Rev	GCCAGGAGCAAGACCGTGTT<br>TGCCCCACCACTGCCTGTA
Uba7	For<br>Rev	GAGTTATACTCCAGGCAGCT<br>CACTGAGCAGCCAAGTCAG
Gbp3	For<br>Rev	ACATGGCCAAATGAAGACACA<br>TGAAAACCCACTTGTGCGTT
Oas2	For<br>Rev	GAAGGATGGCGAGTTCTCTACC<br>GTGCTTGACCAGGCGGATG
Ifit1	For<br>Rev	CAGAAGCACACATTGAAGAA<br>TGTAAGTAGCCAGAGGAAGG
Ifit3	For<br>Rev	CTGAAGGGGAGCGATTGATT<br>AACGGCACATGACCAAAGAGTAGA
Isg15	For<br>Rev	TGACGCAGACTGTAGACACG<br>TGGGGCTTTAGGCCATACTC
Mx2	For<br>Rev	CCAGTTCCTCTCAGTCCCAAGATT<br>TACTGGATGATCAAGGGAACGTGG
Irf9	For<br>Rev	ACAACTGAGGCCACCATTAGAGA<br>CACCACTCGGCCACCATAG
Stat1	For<br>Rev	GAACGCGCTCTGCTCAA<br>TGCGAATAATATCTGGGAAAGTAA
Vdr	For<br>Rev	GATGCCCACCACAAGACCTA<br>CGGTTCCATCATGTCCAGTG
Cyp24a1	For<br>Rev	AAGAGATTCGGGCTCCTTCA<br>GCAGGGCTTGACTGATTTGA
Cyp27b1	For<br>Rev	GCATCACTTAACCCACTTCC<br>CGGGAAAGCTCATAGAGTG
Gapdh	For<br>Rev	AGACGGCCGCATCTTCTTGT<br>ACTGCAAATGGCAGCCCTGG

Table S3: The human specific primers used in this study are listed below

Gene Name		Sequences used for qPCR (5'→ 3')
VDR	For<br>Rev	CTCAAACGCTGTGTGGACAT<br>ACTGTCCTTCAAGGCCTCCT
GAPDH	For<br>Rev	TGCACCACCAACTGCTTA<br>AGAGGCAGGGATGATGTTC

References

1 Olenchock, B. A. *et al.* EGLN1 Inhibition and Rerouting of alpha-Ketoglutarate Suffice for Remote Ischemic Protection. *Cell* **164**, 884-895, doi:10.1016/j.cell.2016.02.006 (2016).

2 Westernströer, B. *et al.* Profiling of Cxcl12 receptors, Cxcr4 and Cxcr7 in murine testis development and a spermatogenic depletion model indicates a role for Cxcr7 in controlling Cxcl12 activity. *PloS one* **9**, e112598-e112598, doi:10.1371/journal.pone.0112598 (2014).

3 Bordignon, J. *et al.* Expression profile of interferon stimulated genes in central nervous system of mice infected with dengue virus Type-1. *Virology* **377**, 319-329, doi:https://doi.org/10.1016/j.virol.2008.04.033 (2008).

4 Dong, H.-J. *et al.* The Wnt/β-catenin signaling/Id2 cascade mediates the effects of hypoxia on the hierarchy of colorectal-cancer stem cells. *Scientific Reports* **6**, 22966, doi:10.1038/srep22966 (2016).

5 Zhao, W. *et al.* Cellular Intrinsic Mechanism Affecting the Outcome of AML Treated with Ara-C in a Syngeneic Mouse Model. *PLOS ONE* **9**, e109198, doi:10.1371/journal.pone.0109198 (2014).

6 Goo, Y. H., Son, S. H., Yechoor Vijay, K. & Paul, A. Transcriptional Profiling of Foam Cells Reveals Induction of Guanylate-Binding Proteins Following Western Diet Acceleration of Atherosclerosis in the Absence of Global Changes in Inflammation. *Journal of the American Heart Association* **5**, e002663, doi:10.1161/JAHA.115.002663.

7 del Fresno, C. *et al.* Interferon-beta production via Dectin-1-Syk-IRF5 signaling in dendritic cells is crucial for immunity to C. albicans. *Immunity* **38**, 1176-1186, doi:10.1016/j.immuni.2013.05.010 (2013).

8 White, C. L., Kessler, P. M., Dickerman, B. K., Ozato, K. & Sen, G. C. Interferon Regulatory Factor 8 (IRF8) Impairs Induction of Interferon Induced with Tetratricopeptide Repeat Motif (IFIT) Gene Family Members. *J Biol Chem* **291**, 13535-13545, doi:10.1074/jbc.M115.705467 (2016).

9 Nacionales, D. C. *et al.* Type I Interferon Production by Tertiary Lymphoid Tissue Developing in Response to 2,6,10,14-Tetramethyl-Pentadecane (Pristane). *The American Journal of Pathology* **168**, 1227-1240, doi:https://doi.org/10.2353/ajpath.2006.050125 (2006).
