# Supplementary material for: An enhanced chemopreventive effect of methyl donor S-adenosylmethionine in combination with 25-hydroxyvitamin D in blocking mammary tumor growth and metastasis
Source: Bone Res. 2020 Jul 22;8:28. doi: 10.1038/s41413-020-0103-6 (PMC7376160; doi:10.1038/s41413-020-0103-6)
Supplement: Supplementary file 2 [file 41413_2020_103_MOESM2_ESM.pdf]

List of DEGs in control vs treated PyMT-R221A cells (log2FC>0.5 and FDR<0.05)

SAM vs Control		
Gene	log2FC	p-value
Ndrg1	-1.334	1.20E-08
Dmp1	-1.206	7.56E-05
Plvap	-1.18	9.63E-05
Eno2	-1.178	0.000143
Pold4	-1.129	0.00233
Mxd1	-1.128	0.000166
Ero1l	-1.107	0.005007
Aldoart1	-1.084	0.000844
Spp1	-1.07	3.99E-05
Ly6d	-1.056	1.91E-05
Prok2	-1.053	0.009601
Trf	-1.045	3.86E-08
Crispld2	-0.982	0.009183
Egln3	-0.978	2.32E-05
Arc	-0.973	0.015649
Selenbp1	-0.967	0.000233
Ankrd37	-0.961	0.011558
Cxcr4	-0.954	0.011802
Pgf	-0.942	2.44E-11
9330188P03Rik	-0.942	0.000143
Cdh23	-0.924	0.008613
P4ha1	-0.918	0.000214
Csrnp1	-0.914	0.002379
Gm17455	-0.914	0.024697
Nptx1	-0.91	0.014361
Fkbp7	-0.905	0.001241
Mgp	-0.897	0.034348
Tnni1	-0.895	0.034962
Ier3	-0.894	3.40E-11
Isg20	-0.868	2.32E-05
Lypd3	-0.866	0.001663
Ace	-0.865	0.023621
2810468N07Rik	-0.864	0.010888
Sept5	-0.862	7.56E-05
Pla2g7	-0.857	0.002458
Tspan5	-0.854	0.000743
Slc2a1	-0.85	0.001746
Tnnc1	-0.85	0.020082
Aplp1	-0.829	2.68E-05
St3gal1	-0.829	0.037943
Gm6402	-0.829	0.046539
Metrnl	-0.821	7.56E-05
4632428N05Rik	-0.818	0.031459
Rps23	-0.817	0.013466
Smtnl2	-0.808	0.003695
Medag	-0.799	0.026638
Cd248	-0.798	0.018472
Bsg	-0.793	0.00018

25(OH)D vs Control		
Gene	log2FC	p-value
Plvap	-1.224	0.005084
Eno2	-1.214	0.013823
Dmp1	-1.193	0.001218
Ndrg1	-1.169	0.004515
Tgfa	-1.15	0.000779
Hey2	-1.118	0.024523
Mir6236	-1.099	0.000405
Mxd1	-1.094	0.016866
Fcgrt	-1.063	0.000447
Tnni1	-1.062	0.035945
Macrod1	-1.055	0.015256
Egln3	-1.049	0.004145
9330188P03Rik	-1.039	0.003441
Lef1	-1.037	0.004647
Selenbp1	-1.017	0.022335
Smtnl2	-1.003	0.016533
Rn45s	-0.984	0.000215
Prss22	-0.982	0.032795
Sema4g	-0.982	0.033666
Espn	-0.982	0.000209
Rras	-0.98	0.024101
Isg20	-0.972	0.002293
Ace	-0.967	0.005476
Sept5	-0.964	0.028902
Vegfa	-0.957	0.004121
Dab2	-0.956	0.029303
Gm17455	-0.955	0.020461
Car9	-0.946	0.002961
Lgals7	-0.943	0.031493
Nptx1	-0.941	0.043165
Cdh23	-0.922	0.045999
Megf6	-0.911	0.000333
Upp1	-0.91	0.037752
Fkbp7	-0.909	0.016561
Mpp2	-0.899	0.019967
Zc3h6	-0.898	0.0491
Gm15867	-0.897	0.020971
P4ha1	-0.892	0.022425
Pglyrp1	-0.87	0.040328
Prelid2	-0.863	0.009449
Fxyd5	-0.86	0.016533
Lypd3	-0.858	0.030098
Mef2c	-0.853	0.005751
Kcne3	-0.85	0.0491
Numbl	-0.85	0.001734
Dhx58	-0.836	0.004029
Plekha2	-0.836	0.027903
4632428N05Rik	-0.832	0.00611

SAM+25(OH)D vs Control		
Gene	log2FC	p-value
Ndrg1	-1.703	2.00E-17
Hey2	-1.597	2.19E-06
Dmp1	-1.531	1.82E-14
Egln3	-1.492	1.54E-22
Rmrp	-1.441	2.654E-05
Cdh23	-1.439	4.18E-07
Eno2	-1.436	2.048E-05
Nptx1	-1.426	3.07E-09
9330188P03Rik	-1.411	1.73E-14
Ace	-1.41	1.61E-14
Mxd1	-1.388	5.28E-07
Cxcr4	-1.386	2.80E-16
Ankrd37	-1.338	3.323E-06
Tgfa	-1.333	3.61E-10
Gm17455	-1.321	9.99E-08
Mir6236	-1.3	4.91E-08
Car9	-1.284	1.13E-15
Tnni1	-1.257	0.0004269
4632428N05Rik	-1.239	3.08E-12
Selenbp1	-1.239	4.078E-06
Ero1l	-1.233	0.0012314
Ly6d	-1.227	1.71E-09
Zc3h6	-1.208	4.463E-06
Bnip3	-1.18	5.31E-11
Tnnt2	-1.161	1.914E-06
P4ha1	-1.155	7.56E-08
Fkbp7	-1.152	7.86E-07
Vegfa	-1.133	8.08E-08
Ank	-1.12	3.09E-08
Lpin1	-1.116	2.11E-14
Smtnl2	-1.109	0.0009434
Tnnc1	-1.107	2.42E-07
Spp1	-1.095	1.004E-05
Csrnp1	-1.082	3.628E-05
Enpp1	-1.079	4.18E-07
Fam13a	-1.073	3.09E-08
Plvap	-1.064	0.0045286
Ocstamp	-1.04	0.0001281
Rgs14	-1.036	0.0073795
Fndc1	-1.028	0.010864
Kcne3	-1.027	0.0004117
Trf	-1.022	1.39E-22
Aldoart1	-1.015	3.902E-05
Pgf	-1.013	3.00E-11
Prelid2	-1.011	2.654E-05
Mpp2	-1.01	9.628E-05
Mef2c	-1.006	2.58E-08
2610528A11Rik	-0.998	0.0138496

Macrod1	-0.792	0.037071
H1f0	-0.791	0.028151
A2m	-0.787	0.039248
Lef1	-0.786	0.030814
Enpp1	-0.786	0.022123
Fcgrt	-0.785	0.000712
Unc5b	-0.783	0.011425
Fam162a	-0.776	0.000109
Tnfaip8	-0.776	0.000712
Hcfc1r1	-0.772	9.21E-06
Rpl37rt	-0.771	0.029648
Aldoa	-0.769	2.41E-05
Rab3d	-0.767	0.000712
Rras	-0.766	0.010484
Rara	-0.76	0.01506
Dguok	-0.756	0.005118
Tmem159	-0.753	0.034962
Cx3cl1	-0.753	0.02556
Ocstamp	-0.753	0.049221
Gpi1	-0.752	1.91E-05
Crlf1	-0.748	0.025546
Sdc4	-0.744	1.69E-06
Ndufv3	-0.744	0.000346
Sgtb	-0.742	0.049551
Tgfa	-0.732	0.039156
Efna3	-0.725	0.020762
Mpp2	-0.725	0.023975
Plod1	-0.723	0.009515
Igsf8	-0.715	0.000143
Fam110c	-0.712	0.037071
Pfkp	-0.712	0.00382
Ppm1k	-0.711	0.048962
Dab2	-0.71	0.035669
Blvrb	-0.709	0.033281
Mrc2	-0.708	0.022467
Litaf	-0.706	0.000361
Ndufa6	-0.7	0.01457
Bambi	-0.697	0.028312
Epha2	-0.693	0.001993
Fam13a	-0.692	0.039248
Bcl3	-0.69	0.020762
Nt5e	-0.689	0.032822
Zc3h6	-0.686	0.029349
Numbl	-0.685	0.004329
Hdac5	-0.683	0.008551
Gm15867	-0.683	0.030222
Msx1	-0.682	0.025971
Rpl28	-0.68	0.012398
Krt18	-0.68	0.000589
Per1	-0.68	0.030257
Rplp2	-0.68	0.033123
Ppp1r18	-0.678	0.035552

Bnip3	-0.83	0.030098
Lpin1	-0.822	0.00507
Fbxl20	-0.822	0.043165
Tnnt2	-0.819	0.013823
Bhlhe40	-0.818	0.006768
Hcfc1r1	-0.817	0.001218
Nrarp	-0.814	0.024523
Pfkp	-0.81	0.043165
Efna3	-0.805	0.0491
Rapgef3	-0.802	0.029658
Aplp1	-0.797	0.004435
Clybl	-0.795	0.012087
Gpr153	-0.792	0.029658
Wipi1	-0.791	0.038236
Enpp1	-0.782	0.048222
Cxcr4	-0.77	0.010736
Bend5	-0.769	0.039504
Tnfaip8	-0.766	0.039744
Ypel3	-0.766	0.002062
Pi4k2b	-0.762	0.039227
Ptrf	-0.761	0.045868
Calcoco1	-0.757	0.019967
Ank	-0.754	0.005397
Tcp11l2	-0.751	0.020681
Tmem205	-0.75	0.016533
Kdelr3	-0.745	0.030098
Rab3d	-0.738	0.017806
Slc2a8	-0.732	0.005084
Fam114a1	-0.729	0.017733
Slc9a5	-0.724	0.008273
Pxdc1	-0.719	0.001867
Mib2	-0.715	0.004321
St6galnac4	-0.707	0.034241
Nyap1	-0.707	0.043409
Ier3	-0.703	0.049211
Tmem243	-0.703	0.041683
Ckap4	-0.692	0.008258
Acsf2	-0.687	0.043005
Tceanc	-0.685	0.031799
Dlg4	-0.684	0.04072
Camk2b	-0.684	0.006835
Fam162a	-0.684	0.024402
Slc27a1	-0.669	0.048062
Cxxc5	-0.666	0.006822
Pdk1	-0.666	0.041729
Bnip3l	-0.665	0.008492
Hdac5	-0.665	0.018145
Krt18	-0.662	0.043165
Mtmr11	-0.661	0.049211
Pcmtd2	-0.66	0.019967
Pgm2	-0.653	0.034806
Trf	-0.645	0.027751

Ccl17	-0.997	0.009306
3110057O12Rik	-0.993	0.0001951
Arhgef9	-0.992	0.0014011
Dab2	-0.987	0.0004691
2810468N07Rik	-0.981	0.000171
Lman1l	-0.968	8.682E-05
Ier3	-0.964	6.736E-06
Fam114a1	-0.962	4.94E-12
Tcp11l2	-0.958	3.48E-07
Isg20	-0.948	0.0008333
Megf6	-0.947	6.58E-08
Fam162a	-0.943	1.02E-10
A2m	-0.935	0.0085134
Unc5b	-0.931	4.001E-06
Cep85l	-0.929	0.0077015
Slc2a1	-0.922	0.0008106
Pfkp	-0.915	9.035E-05
Itgb3	-0.91	0.000384
Medag	-0.895	0.0051659
Lef1	-0.894	0.003544
Sema7a	-0.893	0.0045286
Fank1	-0.89	0.0336123
D630029K05Rik	-0.89	0.0305332
Pyroxd2	-0.89	0.0003916
Lcorl	-0.889	0.004586
Pold4	-0.885	0.0062488
Aldoa	-0.885	7.35E-09
Adamts12	-0.883	0.0053238
Zfp28	-0.879	2.741E-05
Gad1	-0.877	0.0313287
Sema6a	-0.873	0.0063553
Plod1	-0.872	1.85E-11
Pdk1	-0.864	3.174E-06
Hk2	-0.864	0.0108854
Zfp395	-0.863	2.654E-05
Bnip3l	-0.86	3.41E-10
Hdac7	-0.86	0.0104553
Bhlhe40	-0.857	9.035E-05
Ppm1k	-0.857	0.0002572
S100a7a	-0.856	0.0413063
Sgtb	-0.853	0.0265172
Raph1	-0.85	0.0168776
Dguok	-0.85	0.005071
Rara	-0.846	0.0037507
Fam110c	-0.845	0.0011531
6030407O03Rik	-0.844	0.0314309
Rgag4	-0.843	0.0093417
Apln	-0.841	0.0003759
Aplp1	-0.841	0.000434
Hectd2	-0.84	0.02204
Atp8a1	-0.838	4.533E-06
Prr18	-0.836	0.0299649

Anxa2	-0.673	0.002039
Dap	-0.671	0.027195
Ccbl1	-0.671	0.024697
Nrarp	-0.671	0.030257
Rpl31	-0.667	0.027383
Ralb	-0.667	0.020334
Gabarapl1	-0.667	0.022467
D630003M21Rik	-0.666	0.00765
Megf6	-0.665	0.01506
1110007C09Rik	-0.665	0.006083
Vegfa	-0.665	0.003695
Mpzl1	-0.664	0.043558
Camk2b	-0.66	0.001289
Itpka	-0.659	0.036683
Cd82	-0.658	0.041382
Ubtd1	-0.651	0.033554
St6galnac4	-0.648	0.012244
Eya2	-0.647	0.007101
Hdac11	-0.643	0.008452
Rpl23	-0.642	0.026021
Jun	-0.642	0.009515
Dpysl2	-0.642	0.008307
Thra	-0.64	0.039818
Btg1	-0.639	0.028545
Gpr146	-0.639	0.04278
Ptrf	-0.639	0.009592
Epcam	-0.637	0.002152
Lgals3	-0.635	0.029801
Clybl	-0.635	0.01685
Mipol1	-0.634	0.029349
Zfp28	-0.628	0.023621
1110008P14Rik	-0.627	0.024697
Arl3	-0.626	0.005476
Kctd11	-0.626	0.046513
Tcp11l2	-0.625	0.049551
Tbc1d10a	-0.624	0.001241
Sertad1	-0.624	0.036265
Mfge8	-0.624	0.001235
Fam114a1	-0.623	0.039099
Naga	-0.622	0.002744
Hint2	-0.619	0.025788
Mmp17	-0.616	0.029648
Fbln2	-0.613	0.012244
Ing2	-0.612	0.006039
Jmjd6	-0.607	0.034348
Lgmn	-0.606	0.003997
Prelid2	-0.606	0.022123
Map1lc3a	-0.604	0.022467
Kdelr3	-0.601	0.012244
Slc15a4	-0.6	0.019296
Ppard	-0.599	0.040876
Bend5	-0.596	0.047792

Acvrl1	-0.641	0.0491
Zfp354c	-0.639	0.025509
Atp1a2	-0.636	0.0491
Wbscr27	-0.631	0.036366
Mapk3	-0.627	0.034374
Lad1	-0.617	0.011524
D630003M21Rik	-0.608	0.012265
Emp2	-0.599	0.017808
Nradd	-0.596	0.039928
Sdc4	-0.593	0.031
Pddc1	-0.593	0.036366
Tmem106c	-0.592	0.043564
Me1	-0.59	0.024101
Bsg	-0.584	0.038539
Cacnb1	-0.583	0.036727
Elk3	-0.58	0.029303
Pdcd4	-0.58	0.0491
Egln1	-0.576	0.016561
Atp8a1	-0.575	0.03884
Aldoa	-0.573	0.049369
Fstl1	-0.57	0.024523
Itgb3	-0.558	0.009581
Zfp395	-0.557	0.034487
Uevld	-0.554	0.041729
Plod1	-0.549	0.024849
Fbxl4	-0.544	0.045868
Sema4c	-0.527	0.032323
Dap	-0.502	0.024101
Snx19	0.507	0.049211
Hnrnpu	0.514	0.043165
Kank4	0.522	0.030098
Prkar2a	0.528	0.030406
Ndufaf4	0.543	0.049932
Aim1	0.547	0.0491
Gemin5	0.548	0.020461
Pno1	0.549	0.043165
Eif3b	0.553	0.039533
Fam13b	0.557	0.046748
Rpa1	0.559	0.041729
Nsd1	0.562	0.030406
Plscr2	0.568	0.027207
Wdr46	0.579	0.024523
Elac2	0.586	0.03823
Recql	0.587	0.043165
Clspn	0.594	0.024101
Noc4l	0.602	0.044753
E2f8	0.602	0.03823
Hnrnpm	0.604	0.033666
Gpatch4	0.606	0.031634
Nup214	0.606	0.036144
Dysf	0.615	0.017487
Nolc1	0.616	0.00507

D630003M21Rik	-0.83	5.63E-08
Gm15867	-0.83	0.0069402
Atp7a	-0.83	0.0048285
Rdh9	-0.824	0.0336281
Ulk3	-0.824	0.0013557
Gpr3	-0.82	0.0446534
Baiap2l1	-0.819	0.0001701
Sspn	-0.819	0.0141415
Glis3	-0.819	0.022427
Cxxc5	-0.816	3.28E-08
Sept5	-0.811	0.0249808
Bend5	-0.808	0.0002337
Jun	-0.808	0.0019588
Camk2b	-0.806	9.35E-13
Lars2	-0.803	0.0019565
Numbl	-0.803	0.0024626
Tnfaip8	-0.8	0.0004117
Mipol1	-0.799	0.0006842
Scml4	-0.795	0.0069957
Ak4	-0.795	3.07E-09
Zfp354c	-0.794	7.45E-07
Slc39a10	-0.787	2.579E-06
Blvrb	-0.787	0.0084758
Crlf1	-0.787	0.0032017
Clybl	-0.782	0.0130834
Gpi1	-0.78	9.30E-07
Tmem159	-0.78	0.0127286
Tspan5	-0.779	3.69E-07
Me1	-0.778	3.362E-06
Bsg	-0.776	4.73E-08
Anxa2	-0.775	2.026E-06
Tiparp	-0.775	0.0141415
Rab3d	-0.773	0.0010121
Mpzl1	-0.772	0.0014297
Plekha2	-0.771	0.0095416
Itpkb	-0.769	0.0141664
Edil3	-0.768	0.0003554
Sbsn	-0.765	0.021891
Cx3cl1	-0.764	0.02204
Slc46a3	-0.763	0.0322216
Bcl3	-0.762	0.0083954
Atp1a2	-0.759	0.0024626
Nt5e	-0.758	0.016016
Dap	-0.754	7.48E-10
Pla2g7	-0.752	0.0069356
Hdac5	-0.751	4.118E-05
Ccbl1	-0.751	0.006738
Kdelr3	-0.751	0.0093375
Cetn4	-0.75	0.0338127
Dgkd	-0.748	0.001598
Hcfc1r1	-0.745	0.0007161
Btg1	-0.745	0.0003285

Grasp	-0.593	0.04317
Egr2	-0.589	0.010069
Fam96b	-0.588	0.02114
Higd1a	-0.588	0.048359
Sh3bgrl3	-0.588	0.01506
Fam214b	-0.586	0.024898
Bhlhe41	-0.583	0.027195
Mif	-0.582	0.039248
Pfdn2	-0.58	0.026638
Abtb2	-0.579	0.036675
Wipi1	-0.577	0.04793
Dgkd	-0.577	0.029123
Sema4c	-0.577	0.022771
Serinc2	-0.576	0.049241
R3hdm4	-0.575	0.00384
Ypel3	-0.575	0.008868
Ilvbl	-0.574	0.01457
Cdkn2a	-0.573	0.002974
Ppme1	-0.571	0.003215
Ddah2	-0.569	0.041444
Dhrs7	-0.569	0.02034
Snupn	-0.567	0.029801
Arfgap3	-0.567	0.001423
Rpl22	-0.565	0.042304
Atg101	-0.565	0.010287
Ckap4	-0.565	0.003513
Psap	-0.564	0.000589
Itm2c	-0.563	0.004329
Serpinh1	-0.561	0.020065
Emp2	-0.557	0.030257
Cbx8	-0.553	0.040057
Slc35a2	-0.548	0.015505
Trim47	-0.546	0.027195
Aass	-0.542	0.046539
Rps26	-0.541	0.039818
Tmem50a	-0.54	0.028716
Mapk3	-0.539	0.002217
Cd63	-0.534	0.035284
Fam131a	-0.533	0.011734
Ormdl3	-0.531	0.015743
Sil1	-0.528	0.03046
Copz2	-0.527	0.030179
Tpd52l1	-0.525	0.008388
Tmbim1	-0.523	0.008509
Bhlhe40	-0.523	0.007801
Pxdc1	-0.523	0.008551
Trappc4	-0.519	0.046565
Flot2	-0.51	0.007101
Vat1	-0.509	0.039587
Dok4	-0.509	0.037071
Espn	-0.505	0.027207
Proser2	-0.504	0.037071

Mybbp1a	0.617	0.010804
Hn1l	0.617	0.049369
Peg3	0.618	0.031799
Baz1b	0.62	0.031799
Dscc1	0.629	0.0491
Ptcd3	0.637	0.022423
Hsp90aa1	0.638	0.016533
Wdr62	0.643	0.043165
Vars	0.645	0.024101
Thoc1	0.649	0.042509
Cse1l	0.65	0.034487
Wdr77	0.651	0.045124
Slfn9	0.654	0.008862
Ccng1	0.658	0.035123
Polr1a	0.659	0.031
Vac14	0.667	0.039978
Cluh	0.672	0.020053
Nr3c2	0.673	0.041933
Prlr	0.677	0.024101
Kif4	0.685	0.048105
Pfas	0.694	0.031634
Mcm10	0.694	0.0024
Tlr3	0.701	0.010736
N4bp2	0.705	0.005476
Exo1	0.706	0.022425
Nol6	0.707	0.00746
Hsph1	0.707	0.001218
Hirip3	0.718	0.025545
Helq	0.722	0.022335
Pitpnm2	0.722	0.039928
Dido1	0.724	0.036727
Polq	0.726	0.008273
Pmm2	0.726	0.024101
Nr1h5	0.729	0.030406
Exosc2	0.731	0.039928
Aen	0.733	0.017806
Ftsj3	0.734	0.005084
Srcap	0.736	0.001815
Kcnc3	0.738	0.01726
Brpf3	0.742	0.020461
Heatr1	0.746	0.011804
Zfp185	0.749	0.021585
Rad51	0.751	0.0024
Pwp2	0.752	0.024523
Utp20	0.761	0.030406
Mfsd7b	0.762	0.010901
Atic	0.764	0.013295
Dhx9	0.765	0.004029
Mcm3	0.767	0.020014
Slc35g1	0.773	0.023459
Nup155	0.776	0.001205
Cdh15	0.779	0.022335

Rn45s	-0.744	0.0313119
Plcd1	-0.741	0.0424619
Arid3b	-0.74	0.0446534
Hpn	-0.74	0.0108279
Lrrc32	-0.738	0.0367236
Gli1	-0.731	0.0205432
1700025G04Rik	-0.731	0.0001148
Prkg2	-0.73	0.0492829
Egr2	-0.728	0.0136754
Wipi1	-0.727	0.0164002
Egln1	-0.727	4.05E-08
Deptor	-0.723	0.0188528
Slc20a1	-0.722	6.568E-06
Sema4c	-0.721	1.81E-07
Camk2n1	-0.72	5.026E-05
Arl13b	-0.718	0.0313287
Ndufv3	-0.716	1.251E-06
Trappc6a	-0.716	0.0320987
Tmtc1	-0.716	0.0006135
Lad1	-0.713	8.756E-06
Emp2	-0.712	1.28E-08
Acsl4	-0.71	0.0093375
Lpar3	-0.709	0.0024626
Krt18	-0.708	0.0015469
Gcnt4	-0.704	0.0109564
Ss18l1	-0.704	0.014788
Per1	-0.701	0.0299649
Etv5	-0.7	9.035E-05
Zfr2	-0.698	0.0367236
Cd200	-0.697	0.0003576
Col25a1	-0.696	0.0014033
Klf12	-0.695	0.0329978
Inpp5a	-0.695	0.025736
St6galnac4	-0.694	0.004586
Lypd3	-0.692	0.0346651
Arfgap3	-0.69	0.0006789
Rapgef3	-0.689	0.0208997
Mrc2	-0.686	0.0163977
Pgm2	-0.686	0.0004495
Trp53bp2	-0.685	0.0009992
Fbxl20	-0.684	0.0407642
Ypel2	-0.682	0.0034479
Espn	-0.681	0.0299463
Aass	-0.678	0.0003717
Pkp1	-0.678	0.0017762
Foxc2	-0.677	0.0313119
Sertad2	-0.677	0.0205358
Ptger2	-0.677	0.0405889
Slc9a5	-0.676	0.0002571
Gdi1	-0.673	3.338E-05
Rp9	-0.673	0.0163977
Klf13	-0.671	4.421E-05

Hadh	-0.503	0.018472
Recql	0.504	0.026406
Rfx7	0.5084	0.030788
Cspp1	0.5089	0.029894
Plekhh1	0.5097	0.018472
Tmod2	0.5119	0.039248
Zfp407	0.5162	0.022988
Clspn	0.5179	0.016543
Thoc1	0.5203	0.044623
Lin9	0.5268	0.012244
Son	0.5272	0.007837
Dhx37	0.5299	0.034014
Ankrd32	0.5318	0.029349
Mki67	0.5319	0.045327
Dhx33	0.5339	0.002865
Fzd6	0.5377	0.026384
Impact	0.5394	0.029367
Zfyve26	0.5396	0.022123
Mbnl3	0.5434	0.034962
Srcap	0.546	0.002458
Pgr	0.5476	0.037686
Smcr8	0.5489	0.008162
Mpdz	0.5492	0.022123
Fignl1	0.558	0.016543
Mcm10	0.5588	0.004803
Gabpb2	0.5606	0.021191
Wdr76	0.5612	0.019376
Srek1	0.5634	0.039439
Sass6	0.5646	0.016467
Thoc2	0.5655	0.007956
Nup155	0.5661	0.002422
Ap3d1	0.5665	0.02143
Ptcd3	0.5687	0.005476
Wdr62	0.5691	0.02564
Zfp185	0.571	0.03854
Ewsr1	0.5737	0.035533
Trmt6	0.5751	0.039248
Zfp26	0.5752	0.008388
Upf3b	0.5784	0.005476
Phf20l1	0.5786	0.012398
Cdh24	0.5831	0.035552
Chek1	0.5844	0.007801
Cad	0.5847	0.021831
Ttc14	0.5908	0.004329
Zfc3h1	0.5927	0.002871
Ints1	0.5987	0.039248
Helq	0.599	0.043558
Xpo4	0.6031	0.008509
Thada	0.6044	0.021999
Aim1	0.607	7.56E-05
Znf512b	0.6071	0.002268
Wdhd1	0.6109	0.004197

L3mbtl2	0.784	0.043165
Chek1	0.789	0.0024
Mcm4	0.79	0.001734
Ccdc86	0.79	0.015714
Atp7b	0.792	0.041933
Bysl	0.797	0.034808
Mcm6	0.8	0.030406
Nop2	0.8	0.013051
Neto2	0.809	0.005945
Ccne2	0.839	0.006237
Grwd1	0.845	0.049023
Umps	0.846	0.015342
Rbm19	0.849	0.024101
Slc5a6	0.851	0.011923
Sectm1b	0.852	0.03823
Dkc1	0.862	0.047441
Dhx37	0.863	0.005397
Wdhd1	0.864	0.000689
Npr3	0.871	0.00507
Bend3	0.874	0.005397
Camk4	0.876	0.016533
Fasn	0.882	0.035355
Slc39a8	0.882	0.006304
Cdc6	0.892	0.001734
Fancb	0.908	0.029658
Trmt6	0.909	0.005263
Pop1	0.912	0.037752
Scn5a	0.912	0.00608
Slc26a7	0.914	0.029842
Myo5c	0.918	0.002215
Fanca	0.92	0.036727
Trmt61a	0.926	0.030406
Plce1	0.948	0.022335
Ints1	0.986	0.0024
Polr1b	0.989	0.013438
Adam12	0.99	0.043165
Adamts8	0.996	0.000689
Mthfd1	1.012	0.027751
Ogn	1.028	0.038583
2700038G22Rik	1.031	0.043165
Nuak2	1.031	0.043409
Vtcn1	1.053	0.008458
Ccl2	1.059	0.029303
Ano4	1.072	0.031773
Cad	1.077	0.000779
Kcnq1ot1	1.079	0.001218
Kbtbd8	1.102	0.004063
Aldh1a1	1.11	0.007214
Zfp750	1.12	0.000405
Mgam	1.126	0.009074
Lctl	1.134	0.031
Slc38a3	1.142	0.029548

Mxi1	-0.671	0.0351938
Kctd11	-0.671	0.0018334
Chsy3	-0.668	0.0266336
Pcmtd2	-0.664	0.000142
Sbf2	-0.663	0.0457942
Nudt4	-0.662	0.022687
Ralb	-0.662	0.0001952
Sdc4	-0.659	3.385E-05
Tmem263	-0.656	0.004586
Dlg4	-0.655	0.0117899
Lgals3	-0.652	0.0095809
Fyb	-0.649	0.0191588
Ppard	-0.648	0.0196872
Ypel3	-0.646	0.0219166
Chsy1	-0.645	0.0003179
Slc27a1	-0.644	0.010146
Atg10	-0.644	0.0483198
Epha2	-0.642	0.0017762
Mtmr11	-0.641	0.0105685
Gbe1	-0.637	0.033443
Mapk8ip3	-0.637	0.022427
Ilvbl	-0.637	5.105E-05
Tvp23b	-0.636	0.0017282
Plod2	-0.634	3.323E-06
St6galnac6	-0.631	0.0048956
Sertad1	-0.627	0.0206028
Uap1	-0.627	0.0202751
Fam214b	-0.624	0.0002213
Rapgef4	-0.624	0.0205358
Fam63b	-0.623	3.761E-05
Gys1	-0.623	0.0035865
Sh2d3c	-0.62	0.0232146
Tmem205	-0.617	0.0460079
Camta1	-0.615	0.046035
Fkbp14	-0.614	0.0149048
Wwtr1	-0.614	2.85E-09
Bambi	-0.611	0.0156268
Ift43	-0.611	0.0028399
Prrc1	-0.609	0.0004117
Dok4	-0.609	0.0008084
Rpl3	-0.607	0.0167121
Myo1d	-0.607	0.0412096
Tmx4	-0.607	0.0149255
Ldlrad4	-0.605	0.0155667
Bhlhe41	-0.6	0.0384511
Bgn	-0.597	0.0085918
Inafm1	-0.597	0.0229672
Tmem39a	-0.597	0.0242827
Zfp9	-0.596	0.038683
Fbxl4	-0.595	0.0068802
Ddah2	-0.593	0.029635
Csrp2	-0.593	0.006189

Ddias	0.6109	0.048136
Smarcad1	0.6232	0.001006
Casc5	0.6249	0.00571
Wdr81	0.6278	0.008551
Zfp182	0.6295	0.01427
Tug1	0.6306	0.016411
Zfp644	0.6316	0.003635
Hells	0.6381	0.028312
Zfp160	0.6404	0.011425
Pole	0.6455	0.035669
Ccne2	0.6478	0.022008
Rad51	0.6495	0.00765
Fam13b	0.6527	0.000571
Baz2b	0.655	0.001782
Dhx9	0.6609	0.022966
Mmp15	0.6614	0.029894
Ndor1	0.6623	0.035552
Ctns	0.6672	0.016467
Dgcr8	0.6703	0.011567
Piezo2	0.6784	0.037071
Vegfc	0.6852	0.021636
Neto2	0.6966	0.008386
Faxc	0.6984	0.034406
Eif2ak2	0.6985	0.047804
Ccng1	0.6989	0.018339
Fut9	0.7015	0.021329
Rbm12b2	0.7066	0.019972
Gm10033	0.715	0.033123
Gm17066	0.7181	0.009518
D930016D06Rik	0.7191	0.01506
Ccdc149	0.7209	0.033281
4930431P03Rik	0.7241	0.046433
Bend3	0.7291	0.006556
Zfp932	0.7291	0.008666
F830016B08Rik	0.7407	0.047434
Fancm	0.7409	4.92E-05
Tpcn2	0.7423	0.015755
Utp20	0.7466	0.004146
Pcdhb17	0.7491	0.032539
Gen1	0.7535	0.005476
Nsmaf	0.7554	0.023621
Rrp12	0.7569	0.008113
Zfp101	0.7617	0.014538
Polq	0.7657	0.000346
Atm	0.7686	0.015505
Etohi1	0.7702	0.001564
Zfp808	0.7787	0.037071
Brpf3	0.783	0.010262
Yy2	0.7917	0.008386
Xk	0.794	0.014724
Slc35g1	0.7956	0.021049
Prob1	0.7993	0.004329

Shank2	1.157	0.000209
Mal	1.159	0.000405
Pappa	1.167	0.021156
Cyp2f2	1.183	0.016866
Map1a	1.183	0.0024
Krt5	1.269	0.01256
Rrp12	1.292	5.01E-07
D7Ertd443e	1.388	0.000885
Piezo2	1.435	1.67E-09
Dio2	1.441	8.39E-07
Clec12a	1.504	0.001371
Slc16a12	1.534	0.001205
Krt42	1.765	0.000133

Rab3a	-0.593	0.0139878
Txndc16	-0.592	0.010146
Spry4	-0.592	0.0002217
Pak1	-0.592	0.0424619
Uevld	-0.591	0.0002425
Pla2g4a	-0.59	0.0140957
Piga	-0.59	0.0088069
Fbln2	-0.589	0.0028872
Litaf	-0.589	1.513E-06
Map4k3	-0.588	0.0424619
Metrnl	-0.587	0.0017762
Cryz	-0.586	0.0027188
Epcam	-0.583	9.573E-05
Slc41a1	-0.583	0.003544
Ppme1	-0.582	2.657E-06
Sil1	-0.582	0.0016632
Tceanc	-0.581	0.0158129
Ing2	-0.581	0.0003944
Vamp4	-0.581	0.0028825
Itpr3	-0.58	0.0320987
Zfp143	-0.578	0.0282773
Tmc8	-0.578	0.0188901
Fkbp1b	-0.576	0.0435578
Rbl2	-0.575	0.0163977
Fam131a	-0.575	0.0004117
4930523C07Rik	-0.575	0.0424619
Rnf150	-0.574	0.0142207
2810408A11Rik	-0.574	0.0316791
Zfp189	-0.573	0.0115192
Pot1a	-0.572	0.0122299
Ccni	-0.572	0.0378988
Pxdc1	-0.571	0.022427
Kdm3a	-0.57	2.88E-07
Synj2	-0.569	0.0305332
Bcar3	-0.569	0.0033872
Smtn	-0.567	0.0333984
Cdk19	-0.564	0.0396696
Igsf8	-0.563	0.0208596
Naga	-0.562	0.0141415
Eya2	-0.56	0.0120271
Abtb2	-0.559	0.0242827
Ltbp4	-0.558	0.0483198
Calu	-0.555	0.0007535
BC029214	-0.554	0.0173554
Jmjd6	-0.552	0.0108615
Slc35a2	-0.55	0.0006769
Pofut2	-0.55	0.0003916
Ttc3	-0.549	0.0129124
Car12	-0.548	0.0214537
Slc15a4	-0.546	0.0092608
Btbd1	-0.545	0.0305332
Dtx3	-0.544	0.0158129

Ifit2	0.803	0.02187
Srcin1	0.8053	0.027383
Neat1	0.8075	0.035552
Irgm1	0.8111	0.033281
Zgrf1	0.8121	0.002379
Endod1	0.8173	0.030179
Zdhhc23	0.8198	0.02601
Nsd1	0.8297	3.85E-07
Fam169b	0.8304	0.028984
Ifit1bl1	0.8306	0.033123
Cyp2f2	0.8314	0.046666
Pcdhb16	0.8321	0.000993
4933407K13Rik	0.8322	0.027207
Pcdhga8	0.8353	0.032588
Cmah	0.8363	0.049241
Npr3	0.8398	0.00048
N4bp2	0.8399	2.32E-05
Ogt	0.8431	0.002458
Zfp950	0.8497	0.026485
Pcdhga10	0.8508	0.026205
BC005561	0.8527	0.005476
Npb	0.8539	0.049551
Eid3	0.8573	0.049241
Pcdhga4	0.8582	0.04829
Parp12	0.8617	0.039248
Shank2	0.8637	0.001083
9530091C08Rik	0.8639	0.016014
Pitpnm2	0.8685	0.003695
Fanca	0.8735	0.009321
Gdap10	0.8737	0.042331
Rdh10	0.8743	0.034406
Npr1	0.8779	0.019972
Slc6a14	0.8819	0.03864
BC100451	0.8932	0.035552
Spaca6	0.8934	0.014649
Ptbp2	0.894	0.001241
Veph1	0.894	0.025546
F5	0.8969	0.036675
Zfp493	0.8989	0.01126
Exph5	0.9037	0.028984
Samd9l	0.9053	0.034962
Parp14	0.9082	0.032588
Atg9b	0.9092	0.033281
Gbp3	0.916	0.030257
Kbtbd8	0.917	0.001564
Dio2	0.921	0.001737
Clec12a	0.9289	0.028984
Ftx	0.9392	0.002379
Iigp1	0.9481	0.02564
Adamts5	0.9609	0.003855
Trim65	0.9744	0.002999
Oasl2	0.9761	0.019972

Ctdsp2	-0.541	0.0087374
Elk3	-0.54	0.0025422
Scarb1	-0.54	0.0021929
Fchsd2	-0.54	0.0055109
Ndufa6	-0.537	0.0175692
Asph	-0.537	0.0344726
Rnf13	-0.537	0.0019231
Ufl1	-0.536	0.0027602
Mib2	-0.536	0.0316791
Foxa1	-0.533	0.0054692
Hebp1	-0.532	0.0228541
Sec23a	-0.529	0.0297629
Lifr	-0.529	0.0103987
Itm2a	-0.529	0.0135774
Cacnb1	-0.528	0.0167121
Kxd1	-0.527	0.041074
Kmt2e	-0.527	0.0281356
Rlf	-0.525	0.0392112
Mfge8	-0.525	0.0168331
Higd1a	-0.524	0.0155667
Zfp120	-0.524	0.0045363
Trp53i11	-0.523	0.0313162
Copz2	-0.52	0.0073084
Mapk3	-0.518	0.0466006
Rpl23	-0.517	0.0050942
E2f5	-0.516	0.0054311
Wdr48	-0.512	0.033528
Arl3	-0.511	0.0031352
Dock6	-0.51	0.0086455
Eif4b	-0.509	3.362E-06
Lyrm5	-0.508	0.0108415
Itga6	-0.508	6.497E-05
Birc2	-0.508	0.0073084
Dennd5a	-0.507	0.0004084
Nphp3	-0.506	0.0446534
Arl5a	-0.506	0.0058286
Dusp7	-0.503	0.0066991
Cep97	-0.503	0.0205358
Pgr	0.5022	0.0187625
Tgfbrap1	0.5031	0.0063903
Dhfr	0.5052	0.0187264
Rtel1	0.5066	0.0032886
Wdr76	0.5082	0.0045363
Pole	0.5114	0.0407642
Znf512b	0.5134	0.0019943
Upf3b	0.5154	0.0074861
Aunip	0.5168	0.0446502
Shmt2	0.5177	0.0302176
Ndc1	0.5189	0.0055818
Polr3e	0.5199	0.0050942
Dhx33	0.5216	0.002515
Sgsh	0.5224	0.0229672

Adamts8	0.9776	0.002533
Gm8580	0.9782	0.013106
Il13ra2	0.982	0.01506
Cmpk2	0.9835	0.012871
Slfn9	0.9839	8.42E-09
Unc80	0.9845	0.012398
Oas1a	0.9869	0.016467
Malat1	0.9908	0.014468
A930013F10Rik	1.0251	0.011734
Rtp4	1.0339	0.011613
Krt5	1.042	0.010262
Adam12	1.0455	0.004329
Vwa2	1.0496	0.008551
Zfp750	1.0548	0.004393
Ogn	1.0729	0.007101
Scn5a	1.0848	0.000244
Vtcn1	1.0886	0.00047
Mgam	1.1112	0.00302
Tlr3	1.1187	1.91E-05
3110039I08Rik	1.1192	0.004278
Myo5c	1.1288	0.002458
Ifi47	1.1385	0.003695
Pappa	1.2007	0.001306
Trim30a	1.2409	0.001241
Nr1h5	1.2781	0.000221
Tbrg3	1.385	0.000143
Kcnq1ot1	1.5226	1.66E-05

Nat10	0.5253	0.0133009
Ctdp1	0.5259	0.0095697
Mybbp1a	0.5263	0.015023
Zbtb26	0.5267	0.0324206
Pus1	0.5271	0.0174085
Hnrnpm	0.5273	0.04357
Snx19	0.5281	0.0025079
Gemin5	0.5281	0.0057449
Jmjd4	0.531	0.0017673
Mid1	0.5315	0.0343168
Wdr46	0.5316	0.0265133
6530402F18Rik	0.5346	0.0007569
Zgrf1	0.5346	0.0291703
Mgme1	0.5378	0.0179766
Thada	0.5401	0.0056173
Lancl2	0.5429	0.0002165
Mybl2	0.543	0.0175228
Fam132b	0.5445	0.0141415
Clcn7	0.5467	0.0001701
Lrrc14	0.5493	0.040531
Ddias	0.5497	0.0100171
Nkain1	0.5502	0.0014969
Ppat	0.5522	0.0017515
Wdr62	0.5527	0.0284215
Arhgap6	0.5543	0.0362941
Myc	0.5546	0.0405889
Nolc1	0.5617	0.006836
Fjx1	0.5625	0.0080184
Krt14	0.5636	0.0175113
Fzd6	0.5665	0.0017282
Ina	0.5754	0.0068077
Baz1b	0.5762	0.0284425
Chaf1a	0.5843	0.0316791
N4bp2	0.5874	0.0405889
Smg5	0.5885	0.006738
Xpo4	0.5906	0.0205224
Fancm	0.593	0.0142207
Daglb	0.5939	0.0242827
Ap3d1	0.5961	0.0199445
Ptcd3	0.5964	0.0051503
Fgf1	0.5965	0.0225502
Zfp710	0.5967	0.0087493
Heatr1	0.6001	0.0341951
Elac2	0.6014	0.0007113
Exo1	0.6073	0.0225502
Prmt3	0.6078	0.0086738
Hells	0.608	0.0213376
Gsr	0.6083	0.0076368
Cluh	0.6093	0.0123465
Rmdn2	0.6099	0.0164002
Pidd1	0.6121	0.036615
Pfas	0.6129	0.0174893

Cadm1	0.6129	6.52E-05
Gen1	0.613	0.0095416
Plb1	0.6136	0.0182097
Dgcr8	0.6141	0.0316791
Exosc2	0.6164	0.0205358
Ptbp2	0.6165	0.0001132
Ddx21	0.6194	0.0001356
Dhx9	0.6222	0.0282731
Wdr77	0.6237	0.0143453
Eif3b	0.6266	1.364E-05
Aim1	0.6287	0.0001002
Polr1a	0.6291	0.0163977
Rrs1	0.6318	0.0001878
Wdr81	0.6324	0.0154135
Bspry	0.6328	0.0179766
Klhl42	0.6372	0.0246021
Ccdc86	0.6387	0.0181555
Gpatch4	0.6398	0.0016994
Ccne2	0.6418	0.0073795
Me2	0.6431	0.0014011
Bysl	0.6453	0.0371071
Mb21d1	0.6466	0.0016191
Mtr	0.6468	0.0093417
Atp13a4	0.647	0.0189679
Camk4	0.6481	0.039877
Recql	0.6505	3.812E-05
Zfp185	0.6522	0.0247846
Nr2f2	0.6526	0.0045363
Gsg2	0.653	0.0199445
Tmco4	0.6532	0.0048531
Parp9	0.6541	0.0256025
Panx1	0.6549	0.0130186
Cpped1	0.6577	0.0297629
Tbc1d2	0.6582	0.0280575
Tpcn2	0.6627	0.026453
Hr	0.6643	0.0187285
Slc39a8	0.6656	0.0089985
Umps	0.6656	0.0378068
Rbm45	0.6678	0.0088069
Srcin1	0.668	0.035605
Nol6	0.6758	0.0045286
Parp10	0.6761	0.0185519
Ccnd2	0.6802	3.385E-05
Utp20	0.682	0.0244936
Dcn	0.689	0.0240315
D030028A08Rik	0.6933	0.0269041
Prkag2	0.6938	0.0079953
Rbm38	0.6941	0.0164747
L3mbtl2	0.6945	0.0195542
Ftsj3	0.6952	0.0004269
Hirip3	0.6954	0.0045397
Chek1	0.6956	0.0002226

Mcm10	0.6977	0.0004117
Nsmaf	0.6993	0.0380098
Hgh1	0.7029	0.0046728
Recql4	0.7043	0.0001007
Cdc6	0.7073	0.0163977
Uba7	0.7074	0.0353112
Atg9b	0.7079	0.0193632
Heatr3	0.7086	0.0320987
Ints1	0.7086	0.0437746
Rufy1	0.7093	0.0045286
Vac14	0.7097	0.0007841
Ctns	0.7119	0.0004158
Ube2cbp	0.7139	0.0382046
Nop2	0.7216	0.006189
Tapbpl	0.7219	0.0142378
Ttc22	0.7276	0.0251733
Grwd1	0.7284	0.0307301
Slc5a6	0.734	0.0048815
Ubxn8	0.7383	0.0320987
Helq	0.7399	0.001096
Trmt6	0.7436	0.0076983
Neto2	0.7465	0.0042927
Cad	0.7465	0.0391638
Nfatc4	0.7475	0.0297528
Eme2	0.7528	0.0424426
Pitpnm2	0.7541	0.0046711
Prodh	0.7594	0.0365959
Dph2	0.7598	0.0094876
Slfn9	0.7634	2.07E-08
Zdhhc23	0.7639	0.0197882
Sbspon	0.7679	0.0448716
Pramef25	0.7685	0.0492829
Ccdc96	0.7688	0.014826
Mns1	0.7747	0.0200671
Gtse1	0.775	0.0286579
Krt80	0.7761	0.0049451
Ap5b1	0.7765	0.0069356
Atp6v0e2	0.7774	0.0226471
Brpf3	0.7875	0.0002539
Fst	0.788	0.0099885
Pwp2	0.7893	5.904E-05
Sectm1b	0.7905	0.0083917
Mthfd1	0.7915	0.0470855
5430403N17Rik	0.7944	0.0383985
Xk	0.7945	0.0041882
Rad51	0.801	1.28E-07
Ccng1	0.8026	1.004E-05
Shpk	0.8035	0.009484
Trmt61a	0.8055	0.0187285
Fanca	0.8057	0.0272365
Piezo2	0.806	0.0405889
Plce1	0.8119	0.0181702

Slc43a2	0.8122	1.293E-05
F830016B08Rik	0.813	0.0344726
Phf11d	0.826	0.0457942
Klhdc7a	0.827	0.0482769
Pou4f1	0.8289	0.0141415
Gm4951	0.8311	0.0383985
Lrrn4	0.8313	0.0313119
Fam222a	0.8318	0.0092597
Vegfc	0.8329	0.0017515
Plscr2	0.8395	2.19E-06
Slfn2	0.8407	0.0460079
Adgrb1	0.8411	0.0350387
Gm4890	0.8426	0.0405723
Bend3	0.8428	0.0020764
Stat1	0.8447	0.033273
Zbp1	0.8449	0.0378198
Dhx37	0.8478	0.0017515
Oas2	0.8488	0.0195542
Mmp15	0.8499	0.0017515
Polr1b	0.8518	0.0120271
Mir6358	0.8531	0.0469469
Igtp	0.86	0.0419398
Slc24a3	0.8601	0.0114584
Endod1	0.865	0.0069356
Exph5	0.8651	0.015473
Irf9	0.8666	0.0251664
A830018L16Rik	0.8687	0.0297629
Hs6st2	0.8704	0.0004229
Nuak2	0.8725	0.0347459
Atoh8	0.8764	0.0254875
Tdpoz4	0.8769	0.0324206
Robo3	0.8778	0.0390454
Wfdc17	0.8807	0.0352662
Kif26b	0.8824	0.0352136
Eda2r	0.8838	0.0063542
Ism1	0.886	0.0086386
Mx2	0.8894	0.0231602
Usp18	0.8912	0.0192067
Apol9b	0.8924	0.0214537
Pla2g4c	0.8925	0.0313119
Cmah	0.8942	0.0194287
Oasl1	0.8949	0.0225502
Veph1	0.8961	0.0140957
Irgm2	0.8965	0.0336123
Usp17lb	0.8983	0.0313489
Adamts5	0.903	0.000545
Fmo1	0.906	0.0313386
Irgm1	0.9073	0.00856
F5	0.9113	0.016029
Adgrg3	0.9127	0.0244865
Slc35g1	0.9151	1.128E-05
Abcb1b	0.9155	0.006738

Bst2	0.9197	0.0234352
Ccdc149	0.9242	6.788E-05
Ifi44	0.9254	0.0198457
Klk1b1	0.9296	0.0099329
Tprn	0.9297	7.013E-06
Dcc	0.9305	0.0192067
Usp17lc	0.9403	0.0216429
Serpina3h	0.9543	0.0171053
Gdpd2	0.9552	0.0002987
Aen	0.9561	4.12E-08
Bmper	0.9566	0.015777
Slc35f2	0.9633	0.012924
Ryr1	0.9636	0.0076983
Zfp352	0.9699	0.0184044
Iigp1	0.9743	0.0181585
Rnase1	0.9747	0.0085918
Padi1	0.9749	0.0017282
Kbtbd8	0.9765	0.002643
Trim65	0.9821	1.204E-06
Slc6a14	0.9823	0.0169175
Apol9a	0.9942	0.0113083
Oasl2	1.0025	0.0141415
Ifit3	1.0029	0.0088393
Clec12a	1.0045	0.0139883
Irf7	1.0087	0.0135774
Gm4858	1.0096	0.0130939
Rdh10	1.0161	0.0020729
Slc5a1	1.0306	0.0093375
Cd93	1.0334	0.0095416
Npr3	1.0376	8.844E-06
Slc16a12	1.0448	0.0093375
Angptl4	1.0457	0.0089985
Cldn4	1.0505	0.0049707
Msln	1.0548	0.0087176
Ifit1	1.0555	0.0062073
Spon2	1.0557	0.0086386
Mmp10	1.0614	0.0049746
Isg15	1.0629	0.0069356
Ifit1bl1	1.0688	0.0054661
Gm8994	1.0706	0.004615
Parp12	1.0737	0.0016994
Gbp3	1.0772	0.0069356
Adam12	1.0824	0.0014969
Tlr3	1.0938	3.82E-09
Angptl7	1.1017	0.0033921
Mal	1.1026	5.105E-05
Ogn	1.1067	0.0025047
Bbox1	1.1105	0.0033921
Foxj1	1.1167	0.0010942
Vtcn1	1.1315	3.721E-05
Edar	1.1443	0.0030969
Gm8300	1.1496	0.0033196

Oas1g	1.1525	0.0029447
Unc80	1.1542	0.001722
Cdh15	1.157	9.80E-11
Ano4	1.1619	0.0009992
Krt42	1.163	0.0028051
Erdr1	1.1653	0.0022428
Ccl2	1.1793	0.0003535
Oas1a	1.1867	0.0020729
Usp17le	1.1874	0.0012108
Lctl	1.1888	0.0017515
Mmp7	1.1893	0.0015102
Cyp2f2	1.1931	0.0009992
Pappa	1.1938	0.0013208
Rrp12	1.2198	1.28E-08
Rtp4	1.2316	0.000921
Aldh1a1	1.2332	6.736E-06
Them5	1.2377	0.0001768
Dio2	1.2932	2.741E-05
Scn5a	1.2967	3.91E-13
Vwa2	1.3034	0.0001132
Ifi47	1.3054	0.0004602
Myo5c	1.3249	2.38E-07
Mmp24	1.3277	3.812E-05
Zfp750	1.3746	1.89E-11
Adamts8	1.3868	9.90E-12
Papl	1.3879	4.836E-05
D7Ertd443e	1.3994	1.594E-06
Il13ra2	1.4053	0.0001062
Cmpk2	1.4716	4.118E-05
Trim30a	1.4965	2.654E-05
Mgam	1.6127	3.41E-10
Krt5	1.8871	6.72E-09
